# Unveiling the contemporary progress of graphene-based nanomaterials with a particular focus on the removal of contaminants from water: a comprehensive review

**DOI:** 10.3389/fchem.2024.1347129

**Published:** 2024-02-14

**Authors:** Humira Assad, Imtiyaz Ahmad Lone, Alok Kumar, Ashish Kumar

**Affiliations:** ^1^ Department of Chemistry, School of Chemical Engineering and Physical Sciences, Lovely Professional University, Phagwara, India; ^2^ Department of Chemistry, National Institute of Technology, Srinagar, Jammu and Kashmir, India; ^3^ Department of Mechanical Engineering, Nalanda College of Engineering, Bihar Engineering University, Department of Science, Technology and Technical Education, Government of Bihar, Patna, India; ^4^ Department of Chemistry, Nalanda College of Engineering, Bihar Engineering University, Department of Science, Technology and Technical Education, Government of Bihar, Patna, India

**Keywords:** graphene, nanomaterials, functionalization, decontamination, photo catalysis, toxicity

## Abstract

Water scarcity and pollution pose significant challenges to global environmental sustainability and public health. As these concerns intensify, the quest for innovative and efficient water treatment technologies becomes paramount. In recent years, graphene-based nanomaterials have emerged as frontrunners in this pursuit, showcasing exceptional properties that hold immense promise for addressing water contamination issues. Graphene, a single layer of carbon atoms arranged in a hexagonal lattice, exhibits extraordinary mechanical, electrical, and chemical properties. These inherent characteristics have led to a surge of interest in leveraging graphene derivatives, such as graphene oxide (GO), reduced graphene oxide and functionalized graphene, for water treatment applications. The ability of graphene-based nanomaterials to adsorb, catalyze, and photocatalyze contaminants makes them highly versatile in addressing diverse pollutants present in water sources. This review will delve into the synthesis methods employed for graphene-based nanomaterials and explore the structural modifications and functionalization strategies implemented to increase their pollutant removal performance in water treatment. By offering a critical analysis of existing literature and highlighting recent innovations, it will guide future research toward the rational design and optimization of graphene-based nanomaterials for water decontamination. The exploration of interdisciplinary approaches and cutting-edge technologies underscores the evolving landscape of graphene-based water treatment, fostering a path toward sustainable and scalable solutions. Overall, the authors believe that this review will serve as a valuable resource for researchers, engineers, and policymakers working toward sustainable and effective solutions for water purification.

## Graphical Abstract

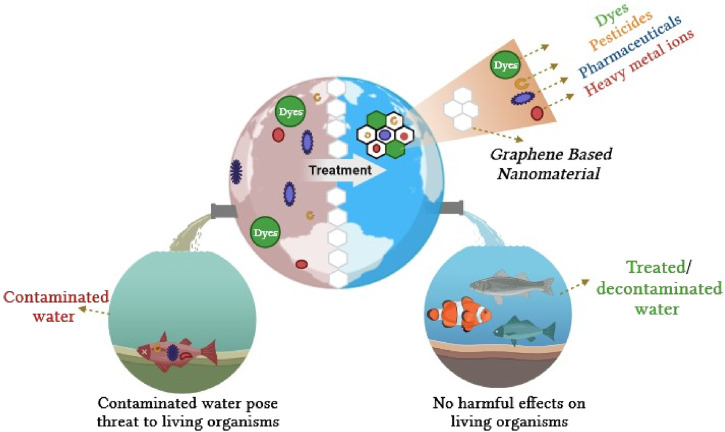

## 1 Introduction

**FIGURE 1:**
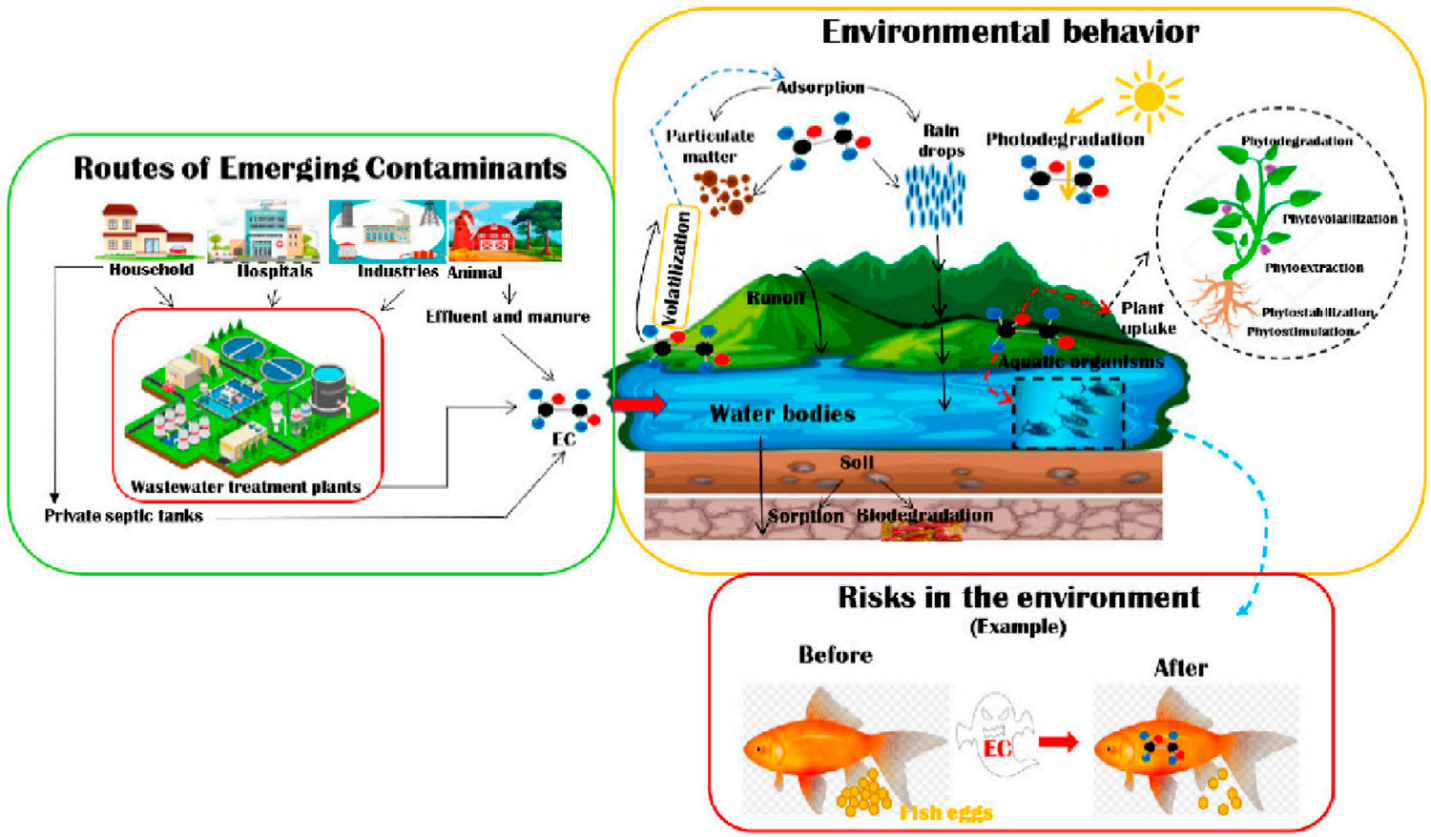
Schematic representation of different entry pathways of contaminants into the environment, their action, and influences (Almeida-Naranjo et al., 2023).

There is a global increase in soil, air, and water pollution due to the world’s rising urbanization and industry (Celik, 2020). A clean and safe environment is therefore essential for survival and maintaining a healthy physique. As water is a vital component of life, water pollution is the most serious form of pollution and has the greatest negative influence on public health and the environment (Mahmoud, 2020). Water has a profound effect on every facet of human existence, such as food, energy, economy, and health (Yin et al., 2020). A fresh water supply is necessary for the protection of children and the impoverished, in addition to the negative effects that inadequate sanitation and water availability have on the environment, the economy, and society (Amin et al., 2014). The textile, pharmaceutical, and metal industries, among others, discharge toxic substances into the environment that damage freshwater bodies. Pesticides, organic dyes, heavy metal ions (HMIs), and other pollutants are among these hazardous substances (Yap et al., 2021). The most hazardous to the environment among these pollutants are HMIs and organic dyes because of their immunogenic, carcinogenic, and mutagenic qualities (Wani et al., 2022). This is because of their low degradability and strong accumulative impacts (Mohd et al., 2022). In humans, animals, and plants, these consequences can reduce neurological, hormonal, and reproductive capacities. When present in appropriate proportions, several heavy metals are considered important nutrients; however, when their concentration surpasses a threshold, they become hazardous to the organism (Lu and Astruc, 2018), as shown in Figure 1.

Waterborne infections are thought to be the cause of 10–20 million deaths annually, while non-fatal infections claim the lives of over 200 million people (Leonard et al., 2003). As per estimates, diarrhea, a water-related issue, claims the lives of 5,000–6,000 children per day (Ashbolt, 2004). Currently, over 0.78 billion people lack safe water supplies globally, which has a negative impact on their health (Amin et al., 2014). The existing water supply will drop by one-third in a few decades, and it is projected that over a billion people worldwide lack access to clean water (Liu and Qiu, 2020). Therefore, a global effort is currently being made to understand how human activity impacts the environment and develop new technologies to reduce any detrimental consequences on public health and the environment in which they live (Gusain et al., 2020). Given that aquifers worldwide are being depleted as a result of various factors, including surface water contamination and saltwater intrusion, now is the ideal time to address water-related issues. Improving purification technology can lessen issues related to energy, water scarcity, health, and climate change (Krishna et al., 2023). Reusing wastewater (WW) can result in a significant reduction in the amount of potable water used, but doing so needs the development of dependable, efficient, and affordable materials and techniques. The burden of micro-pollutants downstream can be reduced by diluting complicated WW effluents, but many of these compounds still pass through conventional water treatment as they exist in micrograms or even nanograms per liter (Verstraeten et al., 2003).

Various treatment technologies have been utilized to eradicate harmful contaminants from water and WW efficiently and comprehensively. Flocculation, membrane filtration, photo-catalysis, chemical precipitation, electrochemical elimination, ion exchange, and adsorption are a few of these methods (Wani et al., 2021). Even though these technologies exist, very few of them are used by enterprises to treat their sewage due to several drawbacks, including high maintenance costs, energy requirements, complex operational procedures, a lack of a circular economy perspective, and sustainability. For the removal of dyes, HMIs, and other hazardous pollutants, the adsorption process is regarded as among the most extensively utilized, valuable, adaptable, and highly efficient techniques available (Khan et al., 2019). Adsorption can be carried out using a variety of materials, such as red mud, fly ash, crop residues, and microbiological cells. Adsorption has several advantages, but despite these, its application in the commercial sector is still quite restricted due to its slow removal effectiveness after a few operating cycles. Adsorbents should ideally have enough binding sites to allow for the effective adsorption of harmful pollutants. If the adsorbent is recyclable, this can further reduce the cost of the adsorption process.

**FIGURE 2:**
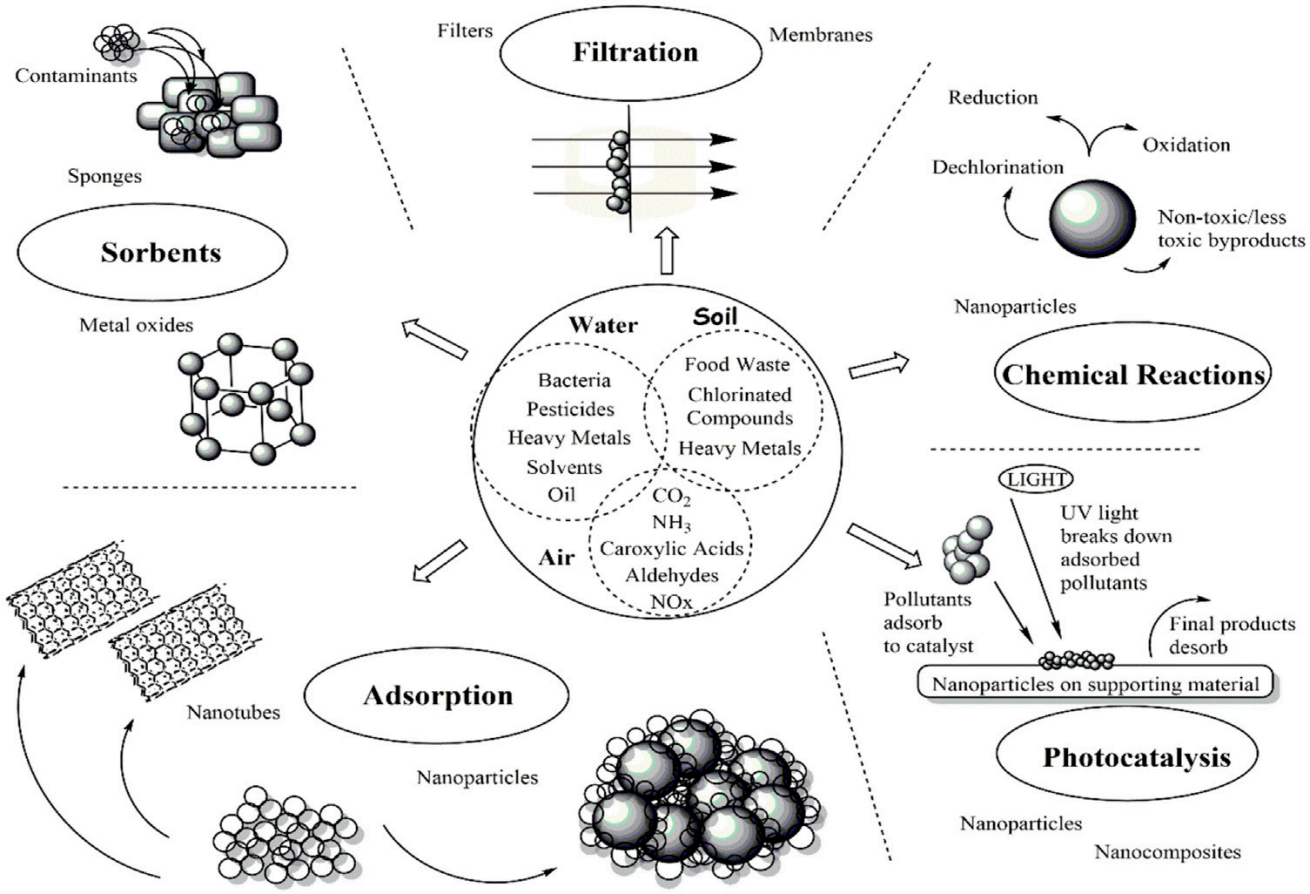
An introduction to general techniques for environmental remediation using nanotechnology (Mathur et al., 2022).

Recent advances in the science of nanotechnology (NT) have sparked considerable attention regarding harnessing the special features of NMs for environmental remediation as one approach to address these urgent environmental concerns (Xu et al., 2012; Assad et al., 2022b). Because of their nanoscale size, NMs have unique properties that can be used to develop new technologies or improve the performance of ones that already exist. A growing body of literature discusses how innovative NMs might be applied to solve significant environmental problems (Assad et al., 2022a). Advanced water systems are developing more effective treatment methods thanks to the use of NMs like CNTs and dendrimers (Kuchi et al., 2021; Ganjoo et al., 2023). To preserve the stability of the ecosystem, NT can be used in numerous ways to address the many water quality issues. The use of NPs and NMs in nanoremediation is the process of eliminating environmental pollutants from contaminated areas. Both chemical and biological processes, including those involving plants, fungi, and bacteria, can produce these NPs and NMs (Nikam et al., 2022). As seen in Figure 2, various studies found that NMs, including GBNs (GBNs), silver (Ag), cerium oxide (CeO_2_), titanium oxide (TiO_2_), zinc oxide (ZnO_2_), nano zero valent iron (nZVI), and nano carbon black (NCB), were effective at removing pollutants (Masindi and Muedi, 2018).

**FIGURE 3:**
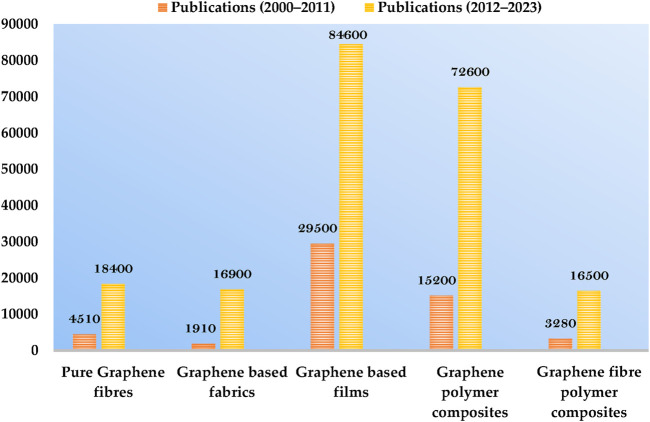
Graphical representation of the growing number of publications on graphene-based materials. The bars represent the number of publications retrieved from Google Scholar. The examined time interval was (left) 2000–2011 and (right) 2012–2023.

Graphene, a 2D material comprised of layers of carbon atoms that create six-membered rings, is considered the ultimate graphitic form (fullerenes, CNTs, *etc.*), Zhi and Müllen (2008) and has caught the attention of scientists. The unusual physicochemical characteristics of graphene, particularly its extraordinarily high SA, ē heat mobility, and mechanical potency, are what initially sparked interest in it Assad et al. (2023c). Theoretically, graphene has the largest specific surface area (SA) of any substance at 2,630 m^2^ g^-1^ and is considered the perfect material for adsorption or surface reaction processes (Perreault et al., 2015). In addition, graphene provides good support for securing chemical functions or NMs; as a result, GBNs and nanocomposites have attracted a lot of consideration from researchers looking for new materials (Al Faruque et al., 2021). The total number of publications retrieved from Google Scholar for the past 2 decades is shown below in the chart graph (Figure 3), in which the gradual increase in the use of graphene-based materials is clearly discernible.

Graphene or graphite can be employed as the initial material for making GBNs, which are graphene-like nanostructures. They differ from each other due to surface chemistries, the quantity of imperfections, and lateral dimensions yet both include sp^2^ and sp^3^ hybridized C atoms. Graphene oxide (GO), reduced graphene oxide (rGO), and graphene quantum dots (GQDs) are examples of GBNs (Lúcio et al., 2021). Moreover, graphene-based nanomaterial production and application-related articles and patents have grown rapidly and continue to do so (Zou et al., 2018). Unique characteristics of GBNs have been discovered, and new processes for the quick and effective fabrication of graphene-based nanocomposites with applications in numerous domains have been covered extensively in review articles over the past few decades (Ghany et al., 2017; Ganjoo et al., 2023). Molina et al. examined the most important studies concerning graphene-based electrochemical sensors for the measurement of toxic ions (Molina et al., 2016). Contemporary progress in the preparation and use of GBNs for drug delivery were reviewed by Wang et al. (2017). Additionally, Madni et al. (2018) examined the biological and physicochemical features of GBNs and described the approaches for producing them. Jiang et al. (2019) created a flexible LED employing laser-induced reduced graphene oxide (LI-rGO), which has a wall plug efficiency (WPE) of 1.4% and a luminous endurance of over 60 h.

In actuality, materials based on graphene provide a wide variety of opportunities for restoring the environment and using electricity. Furthermore, numerous 2D graphene NMs, including pure graphene, GO, and reduced GO, have been developed as important crucial NMs for decontaminating soil (Fathizadeh et al., 2017). These nanoparticles are well renowned for their prowess as adsorbents and antifouling experts. They also possess PC qualities. When compared with ordinary polyamide membranes, graphene membranes have nuclear-level fineness, which increases filtering (Homaeigohar and Elbahri, 2017). The tendency of graphene to form prolonged aggregates or even regain graphite stacking and van der Waals interactions represents the biggest barrier to deploying GBNs in the realm of environmental protection, which results in insufficient separation between the sheets (Khalid et al., 2020). Toxic contaminants can be removed from WW with the use of graphene-based nanoparticles, as evidenced by the numerous excellent evaluations that have been published to date. However, the majority of these assessments concentrate on a single class of pollutant or are restricted to a specific kind of material or adsorbent. We have covered in-depth the adsorption and photodegradation of different pollutants, such as antibiotics, pesticides, dyes, and HMIs, on graphene and GBNs in this review. These processes are essential for the effective use of GBNs to treat contaminated WW. In the meanwhile, an evaluation of the materials' risks, as well as the difficulties and prospects of getting rid of the pollutants that have been clarified, are presented to bring about additional fascinating advancements in this relatively new but extremely promising subject in the future. By concentrating on the difficulties facing future research, the authors hope that this work will offer a distinctive viewpoint on the fundamental studies of GBNs for the management of water and WW.

## 2 Fabrication methods

**FIGURE 4:**
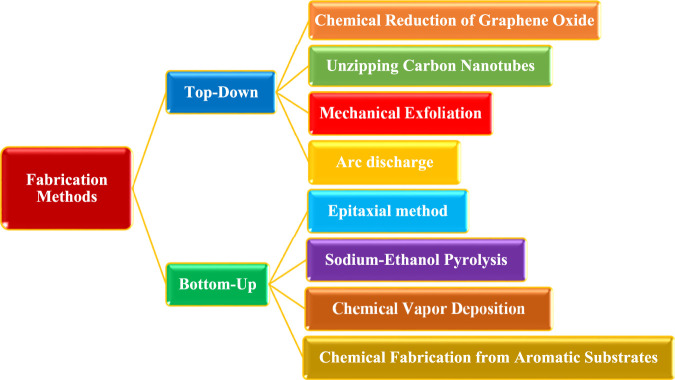
Schematic representation of fabrication methods of GBNs.

It would be ideal if the fabrication of graphene and materials based on it could be managed to give rise to features that would be useful in particular contexts. As is well known, there are two primary approaches for fabricating graphene: top-down and bottom-up (Jana et al., 2017). Top-down methods require the segregation of assembled graphite sheets to produce solitary graphene sheets, while the bottom-up process brings the production of graphene from different carbon sources (Yang et al., 2016), as shown in Figure 4.

How to generate effective substance in a repeatable means, on a huge level, and at a reasonable price is one of the key difficulties in commercializing graphene. Although this is still a significant difficulty, several other ways to make graphene have been explored recently. Given that a significant portion of graphene-based filters are crafted using top-down methodologies (Li et al., 2021), this section will exclusively elucidate the top-down approach and its associated techniques. By tearing apart the stacked layers of graphite, this technology uses procedures to create a single graphene sheet (Sengupta and Hussain, 2019; Vesel et al., 2019). Moreover, graphite or other C-sources, like CNTs (carbon nanotubes), fullerenes, or bigger graphene sheets that are sliced into minor monoatomic C bits, are the starting point for top-down techniques for creating GBNs (Jana et al., 2017; Pedrosa et al., 2020). These techniques could be physical, chemical, or mechanical. The next section discusses a few top-down strategies.

### 2.1 Mechanical exfoliation from graphite

The first high-quality graphene was produced utilizing scotch tape and mechanical exfoliation from raw graphite (Sahu et al., 2021). The original two-dimensional atomic lattices of graphene are completely preserved despite the method’s primitiveness and low efficiency (Dasari Shareena et al., 2018). This technique includes regularly rubbing SiO_2_/Si substrates with scotch tape to exfoliate graphite (Jana et al., 2017). Single or few-layer graphene of very good quality can be distinguished with realistic discrimination thanks to the distinctive color contrast, despite being laborious and poor yielding (Chang and Wu, 2013). The extensiveness of mechanically exfoliated graphene means ultrahigh mobility and a variety of fascinating electrical characteristics are easily observable (Geim, 2009). A great deal of attention toward GBNs and other 2D atomic crystals was sparked by the effective mechanical exfoliation of the first high-worth graphene from graphite (Chang and Wu, 2013).

### 2.2 Unzipping carbon nanotubes

Unzipping carbon nanotubes is another technique that is frequently used to investigate the physics of graphene because of its comparatively good quality. Oxidized graphene nanoribbons were produced by longitudinally slicing multi-walled CNTs employing laser irradiation, plasma etching techniques, or wet chemical methods (Sahu et al., 2021). This unlocking results in the production of graphene nanoribbons. The nanoribbon widths are determined by the tube diameter. After immersion in H_2_SO_4_, the nanotubes were cut and treated in KMnO_4_ (Sahu et al., 2021). Uneven sides emerge from CeC fission, which is often started at the distortion sites to decompress the nanotubes (Cho et al., 2011). Owing to the existence of oxygen deformation sites, the resulting graphene nanoribbons are strong conductors but have a lower electrical quality than commercial-scale graphene layers (Kosynkin et al., 2009). Later, the unzipping of flattened CNTs, where outbreak arises beside the bent edges, was carried out (Saeed et al., 2020) to generate nanoribbons with smooth edges.

### 2.3 Chemical reduction of graphene oxide

An alternative method of making graphene is the reduction of GO (De Silva et al., 2017). The presence of polar O and OH groups causes the graphite oxide to become hydrophilic throughout the oxidation process (Pei and Cheng, 2012; Dasari Shareena et al., 2018). Water is one of the solvents used in the chemical peeling process of this GO. Numerous graphite oxide nanoplatelets are produced by the sonicated graphite oxide solution (Li et al., 2021). The reduction process involves the removal of oxygen units using reducing chemicals. Stankovich and others employed this method by considering a hydrazine-reducing agent; however, it was found that the reduction advancement was insufficient, leaving some residual oxygen. When graphene is being manufactured, GO is created as a precursor (Stankovich et al., 2007). GO is advantageous over graphite due to its hydrophilic behavior (Pedrosa et al., 2020). Sonication is used to suspend GO in water. After that, spin coating or filtering is used to deposit it onto surfaces to create single/double layer GO. Then, to create graphene films, this GO is reduced by thermal or chemical actions (Marcano et al., 2010). The methods (Sieradzka et al., 2021) used to create GO include.

• Wet chemical synthesis
• Plasma functionalization
• Radio frequency plasma

### 2.4 Arc discharge

This process creates a few layers of graphene by applying direct current under high pressure from hydrogen gas between electrodes constructed of ultra-pure graphite (Li et al., 2021). Shen et al. found that the combination of helium (He) and hydrogen (H_2_) gas accounts for the material’s remarkable crystallinity out of all the different gases considered (Shen et al., 2012). The development method comprises a progressive route of graphite evaporation and reactive-gas-restrictive crystallization of the vaporized carbon units. Aqueous arc discharge technology, according to Kim et al., can generate bi- and tri-layer graphene (Kim et al., 2016). With the aid of a temperature increase, heat transfer, and aqueous turbulence, graphene was extracted from the graphite electrodes. A median proportion of graphene sheets with fewer oxygen-related defects were created by varying the voltage of the arc discharge (Li et al., 2021). Owing to a lack of scalability, intricacy, and the high expense of the current technique of synthesizing graphene, GBN research is still a long way from having any important uses.

## 3 Structure and characteristics of graphene-based NMs

**FIGURE 5:**
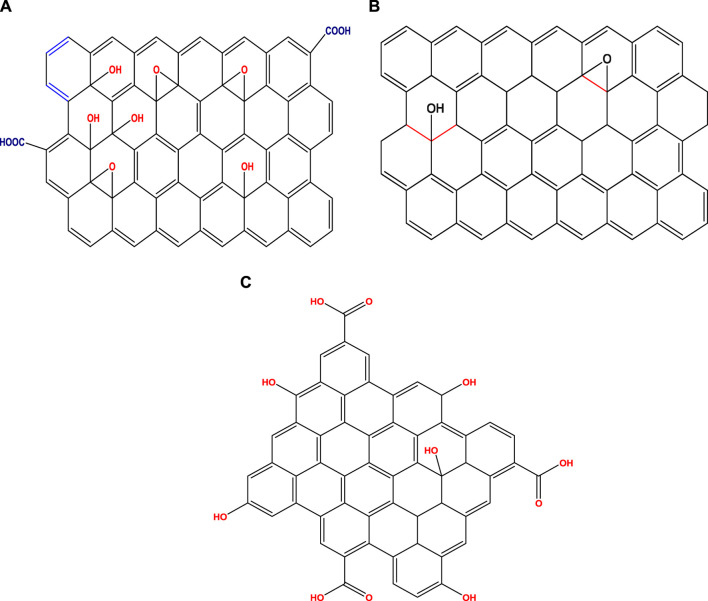
Schematic depiction of several graphene variants. **(A)** Graphene **(B)** Graphene oxide **(C)** Reduced graphene oxide.

Graphene is regarded as the basic component of the family of carbon-based substances, as it can roll into 1D CNTs, wrap into 0D fullerenes, and stack into 3D graphite, enriching the family of carbon compounds. GBNs are usually classified based on layers, oxygen-containing group matter, and other chemical components. According to one study, the structural variations of GBNs, which control their physical and chemical characteristics, are numerous (Yang et al., 2018a). Graphene is criticized as an “impractical material" as it has not been demonstrated that monolayer graphene is stable, even though past research on GBNs concentrated mostly on its configuration and capabilities. Landau, who asserted that rigid 2D crystals are thermodynamically impossible, was proven wrong by the planar 2D framework of graphene (O’Hare et al., 2012). Furthermore, it was discovered that graphene is not a perfect 2D crystal using analysis methods like Raman spectroscopy, TEM, and AFM. To improve stability, graphene sheets are distorted. The chemical makeup of GBNs is depicted in Figure 5.

Standard graphene is a monolayer of graphite with a hexagonal planar organization of C-atoms. With a reported width of approximately 0.335 nm, graphene is the reediest 2D constituent ever to be found. It was initially produced using CVD to mechanically or chemically exfoliate graphite (Zhao et al., 2017a). Three identical carbon atoms are linked to one carbon atom. A C–C bond is a σ-bond created by sp^2^ hybrid orbitals and recognized by its bond length and bond angle, which are approximately 1.42 nm and 120°, respectively. A carbon atom contributes one electron to the system by joining the p orbitals of its neighboring carbon atoms. As п-bonds and a cloud of ē pairs are honeycombed, graphene’s skeleton is made of them. Identical in structure and functionality to graphene, GO also possesses outstanding physiochemical qualities. GO is a single-layer form of graphene that is created when powerful acids and oxidants react. The outer layer of the material encompasses several functional moieties, including -OH, -O, and -CH (O) CH-. In contrast to graphene, the GO structure uses a very specific approach for the oxygen atoms' bonding with the carbon atoms. In addition to having a monolithic configuration akin to graphene and an extremely selective surface area as a consequence of the oxidation reaction, GO also has several functional units on its upper surface that make it simpler for it to endure additional surface reconfiguration. This is the main driver behind GO’s extensive adoption in a variety of industries. GO simultaneously possesses sp^2^ and sp^3^ hybrid orbitals, which stand for the aromatic and aliphatic domains, respectively. GO reduces the conductivity of graphene, but reduction can restore it (rGO). A thermal or chemical reduction of graphite oxide or graphene can result in the intergradation known as rGO (Cheng et al., 2020). Between the perfect sheets of graphene and extremely oxidized GO, rGO is regarded as an intermediary structure (Reina et al., 2017). By using a reducing procedure, such as chemical and physical reduction procedures, the oxygen units on the interface of GO can also be lowered in the case of rGO. Moreover, owing to the lowering of faults brought on by the inclusion of functional groups (FGs), such reduction aids in strengthening the graphitic nature of GO. Moreover, GQDs, a most recent variety of GBNs, are often created utilizing artificial processes including thermal plasma jets. They are thinner (1–20 nm, less than 10 layers), have carbon bonds that are similar to those in graphene, have a greater SA, and splicing that is appropriate for surface modification (Zhao et al., 2017b). Additionally, owing to the edge effect and the same quantum limitations as the C point, GQDs display a novel phenomenon known as steady PL. GQDs have hydrophilic groups like -OH and -COOH at their edges, which enable researchers to alter them as required (Li et al., 2018; Chung et al., 2021).

Since 2004, numerous intriguing characteristics of graphene, including its high SA, great electrical and mechanical conductivity, and outstanding thermal characteristics, have been identified. There are many resources of knowledge concerning the characteristics of graphene (Soldano et al., 2010). Here, we emphasized how the exemplary structure of graphene and GBNs are related to the unique honeycomb pattern of graphene and its products. We concentrated on the characteristics of graphene and GBNs that are of utmost importance for their implementation in a variety of domains, such as biomedicine, the environment, and industry.

### 3.1 Mechanical properties

Each C atom in a graphene layer is covalently connected to three nearby atoms as a result of the 2D honeycomb configuration of carbon atoms. A single defect-free graphene sheet is therefore approximately 200 times mechanically extremely durable due to the tight C-C covalent connections that give graphene its exceptional structural stiffness. This explains the remarkable mechanical properties of graphene, which include its 1 TPa Young’s modulus, 0.149 GPa Poisson’s ratio, and 130 GPa fracture strength (Lee et al., 2010). Owing to its remarkable mechanical qualities, interest in using graphene as a filler to improve the tensile strength of lighter substances has grown (Potts et al., 2011). In comparison to graphene, the surface moieties and imperfections left behind from oxidation or other handling operations have a considerable impact on the mechanical characteristics of GO and rGO. However, these GBNs still have a very high stiffness. These NMs may be employed to fill or reinforce the frameworks of medical apparatuses, hydrogels, biodegradable coverings, electrospun fibers, and other tissue engineering scaffolds because of the amazing mechanical stability of graphene and the good mechanical qualities of GBNs (Zhao et al., 2020). When juxtaposed with CNTs, graphene can dramatically improve the characteristics of polymers that have also been thoroughly examined as nanofillers for polymer matrixes. This is because the large SA of the planar graphene layers permits better relationships between the sheets and the polymer matrix (Perreault et al., 2015).

### 3.2 Physicochemical properties

The enormous SA and sp^2^ network of graphene are its primary unique physicochemical properties. These two qualities give graphene a high degree of reactivity. The electrophilic replacement processes that the graphene planar and ē arrangements can participate in include click reactions, cycloadditions, and reactions to carbine insertion. Additionally, the sp^2^ network allows for п-п stacking interactions with aromatic compounds seen in biomolecules or pharmaceuticals. Finally, while the hydrophobic character of pure graphene is indicated by its water contact angle of 95°–100°, medicinal substances may potentially create hydrophobic connections with graphene through van der Waals interactions. Owing to graphene’s strong hydrophobicity, it is difficult to disperse it in aqueous environments, necessitating the addition of stabilizing agents such as surfactants to prevent clumping in biological fluids (Goenka et al., 2014). The hydrophobic unaltered regions of graphene that are preserved by GO can form п-п interactions that are suitable for drug loading and non-covalent functionalization. Nonetheless, it may be claimed that GO has a higher loading potency as it has more hydroxyl and epoxide moieties that can interact weakly with other groups of medicinal drugs and create hydrogen bonds with them. As additional oxygen functionalized entities in GO are ionized at specific pH levels (for instance, at pH values >4.5, carboxyl FGs are negatively charged), GO also exhibits an amphiphilic character. The reactivity of GO is increased by the occurrence of ionizable moieties and negative charges because more electrostatic interactions with therapeutic substances can be generated. Additionally, charged groups decrease GO’s water contact angle to 30.7°, increasing aqueous dissolvability and, as a result, colloidal stability (Zhao et al., 2017a). As opposed to graphene, rGO has more flaws that occur during the oxygen removal process in GO, rendering it more hydrophobic and less reactive than GO.

### 3.3 Thermal properties

Graphene has high thermal and electrical conductivity due to the п-п bonds beneath and above the carbon atomic plane. In actuality, the C atom typically has 4 ēs available for reactions; however, in graphene, each atom is given one unbound ē that may move randomly across the crystal framework, resulting in exceptionally good TC (TC) (Balandin et al., 2008). Therefore, it has been noted that defect-free graphene has a TC of between 4,500 and 5200 W/mK (Balandin et al., 2008). Manufacturing flaws in GO and rGO break the sp^2^ orbitals of graphene and add a lot of outer layer moieties that block heat transport, lowering the TC of these GBNs (Goenka et al., 2014; Zhao et al., 2017a). Even though increased TC has been useful for many purposes, it is not necessary for all of them. Offering greater thermal insulation features, such as flame retardants and in-house insulation, might be advantageous in some circumstances. It has been found in several recent studies that GO is a useful filler that can improve the flame-retardant qualities of several PNCs. To create superinsulation flame retardant foam, the scientists tried to create CNFs by combining GO oxide and sepiolite clay nanorods. The TC of the produced films has been observed to be 15 mW m^-1^K^−1^ (Zhang et al., 2016). For various application needs, maintaining heat conductivity has therefore been crucial for GO materials.

## 4 Functional modifications of graphene-based NMs

Owing to their superior performance, GBNs have seen extensive use; nonetheless, unaltered GBNs still have several drawbacks. For instance, graphene is extremely hydrophobic, which harms how well it disperses in water. Owing to the charge-defensive properties of the surface moieties, GO tends to assemble in the physiological environment (Jiang et al., 2020). GO has a potent protein-adsorption action and is quickly identified and absorbed in living tissue by macrophages, resulting in inflammation (Kumari et al., 2020). Additionally, in biomedical applications, GBNs lack *in vivo* targeted, delayed, and controlled release capabilities. These flaws collectively restrict the use of GBNs in several domains, especially in biomedicine. The functional modulation by the outermost layers functional of GBNs is a significant method for improving their biological functions as it promotes the stability and water solubilization of GBNs and offers them advanced features such as directed, slow, and controlled release. Covalent and non-covalent alterations make up the majority of surface changes nowadays.

Productive double bonds, polymeric materials, and characteristic FGs are just a few examples of the groups that can be added to the surface of GBNs through covalent modification. Amidate, free radicals, and other chemical methods are used in acidic environments to interact chemically with the active surface FGs of GBNs and produce covalent bonds, which subsequently confer the required activities. Owing to the plentiful FGs that contain oxygen and their potential for covalent modification, GO is the primary method employed to modify GBNs. The possibility of using GBNs for drug delivery, imaging, and diagnostics is increased by covalent modification, which results in fewer electron networks. Additionally, the structure of GO may change in a high-acid environment, improving its physical and chemical properties. Utilizing free radical interactions, Peng and coworkers created a functionalized styrene copolymer alteration of graphene (Peng et al., 2017). The outcomes showed that the distribution and conducting properties of graphene were appreciably improved by free radical transplantation *via* polymerization. Covalent alteration of GBNs may also lead to a faster and more efficient release of drugs at the malignant tumor site, leading to more precise and effective therapy. GBNs that react to stimuli include those that are glutathione, light, heat, and pH responsive. Additionally, small molecules can be used to covalently functionalize GO, leading to new molecular identification techniques for the creation of GBN-targeted formulations. The manufactured Fa-GO may maintain enduring uniform diffusion and steadiness in physiological solutions by covalently grafting folic acid (Fa) to active GO using SO_3_H units, for instance. Additionally, the drugs loaded on GO can be directed at tumor sites exaggerating folate receptors. To create the multifunctional GO that is so vitally needed in biomedical sectors such as drug delivery, biosensing, imaging, tissue engineering, and photo-thermal therapy, this covalent version of GO is especially appropriate for coupling with biomolecules (nucleic acids and others).

On the other hand, the non-covalent modification strategy relies on non-covalent forces to achieve the goal, including ionic and hydrogen bond, van der Waals, п-п, electrostatic, and coordination interactions between the changed moieties and GBNs (Li and Papadakis, 2020). Interestingly, the units of GBNs have a very high degree of aversion to H_2_O molecules, with clear van der Waals bonds and п-п layering, which makes the requirements for the non-covalent transformation of these molecules quite simple. In general, GBNs can undergo non-covalent modification through surface absorption or polymer/biomacromolecule encapsulation. The capabilities of GBNs for dispersion, safety, reactive activation, and biosensors are improved by non-covalent modification. Non-covalent modification does not create chemical bonds; therefore, it has a weaker force of modification, less stability, and is more susceptible to environmental influences than covalent alteration. As a result, *in vitro* and *in vivo* non-covalently modified GBNs are less stable. The active unit or architecture on the outer layer of GBNs is not compromised, and the structure and characteristics of the GBNs are completely preserved. GO (PVA-GO) was functionalized by Chen et al. using covalent and non-covalent methods (Chen et al., 2018). The findings revealed that compared with covalently altered PVA-GO, non-covalently modified PVA-GO had fewer strata and a lower defect concentration while still maintaining all of graphene’s inherent properties. As it can adsorb organic and inorganic components, п-п bonding is the most efficient non-covalent alteration technique. According to a previous study, gold nanoparticles, naphthalene, phenanthrene, and porphyrin complexes with amantane grafts can all form non-covalent bonds with graphene and GO (Sun et al., 2019). By generating stable GO colloidal suspension, which has the potential to be engaged as a carrier substance for biomedical purposes, the active units on GO mix with other functional moieties quickly and efficiently without the presence of contaminants.

## 5 Removal of water contaminants by graphene-based nanomaterials

**FIGURE 6:**
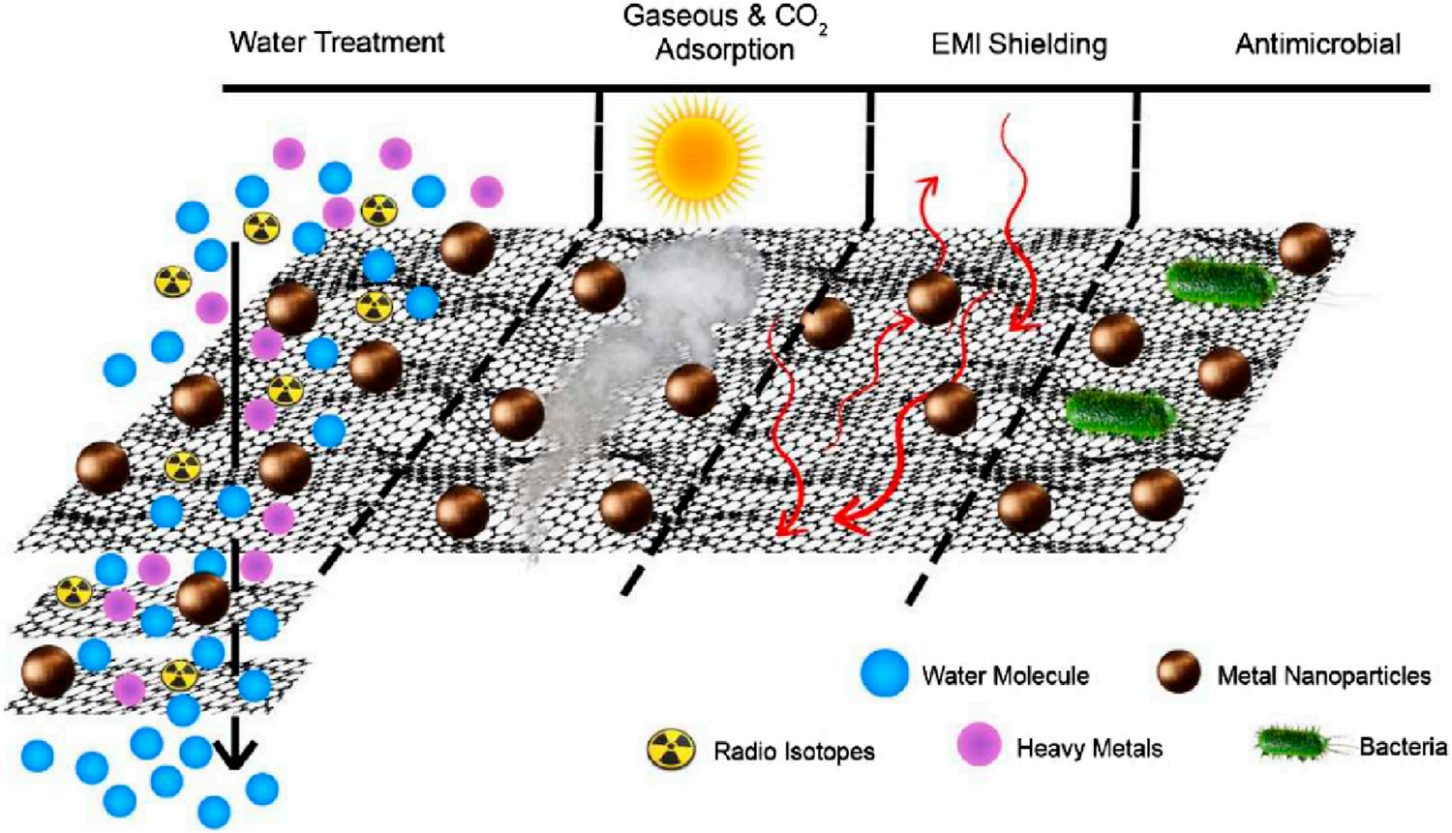
Diagrammatic representation of graphene oxide hybrids used in water filtration for environmental applications (Joel and Lujanienė, 2022).

Because of their exceptional structural and functional characteristics, GBNs are used in a huge range of cutting-edge applications (Yang et al., 2017b). The intensive study of graphene over the past few decades has led to its widespread use in industries ranging from aircraft to agriculture. Owing to the numerous applications of NMs based on graphene, many areas of research have undergone a revolution. It has piqued a broad array of attentiveness and acknowledgment primarily because of its promising prospective applications in fields of research such as metal-eradication sensors and nuclear waste optimization (Jaiswal et al., 2018). The quantity of pollutants discharged into the environment has dramatically increased as a consequence of the rapid population rise and strengthening of agricultural and industrial operations. These extremely diversified pollutants pose a stern threat to environment and general health (Adel et al., 2022). As a result, there is an international effort underway to create reliable technologies that can efficiently remove toxins from the air and H_2_O. Adsorption is a rapid, low-cost, and efficient technique for removing toxins from aquatic habitats among these approaches (Tara et al., 2021). Through physicochemical interactions, the contaminant (adsorbate) is bound to the nanomaterial (adsorbent) during the adsorption process. The use of graphene-based materials has sparked studies at the intersections of many fields, notably environmental restoration, as shown in Figure 6.

GBNs have several π bonds, and GO has numerous FGs with oxygen that are helpful. Every material based on graphene has a large surface area. Five possible interactions might occur when GBNs are adsorbed together: hydrophobic, electrostatic, covalent, and hydrogen bonding (Zhu et al., 2010). Graphene-based materials work well as adsorbents for a variety of waterborne contaminants. In this section, we have briefly elucidated the use of NMs based on graphene as adsorbents to confiscate inorganic and organic contaminants from H_2_O, as elucidated below.

### 5.1 Removal of heavy metals

**FIGURE 7:**
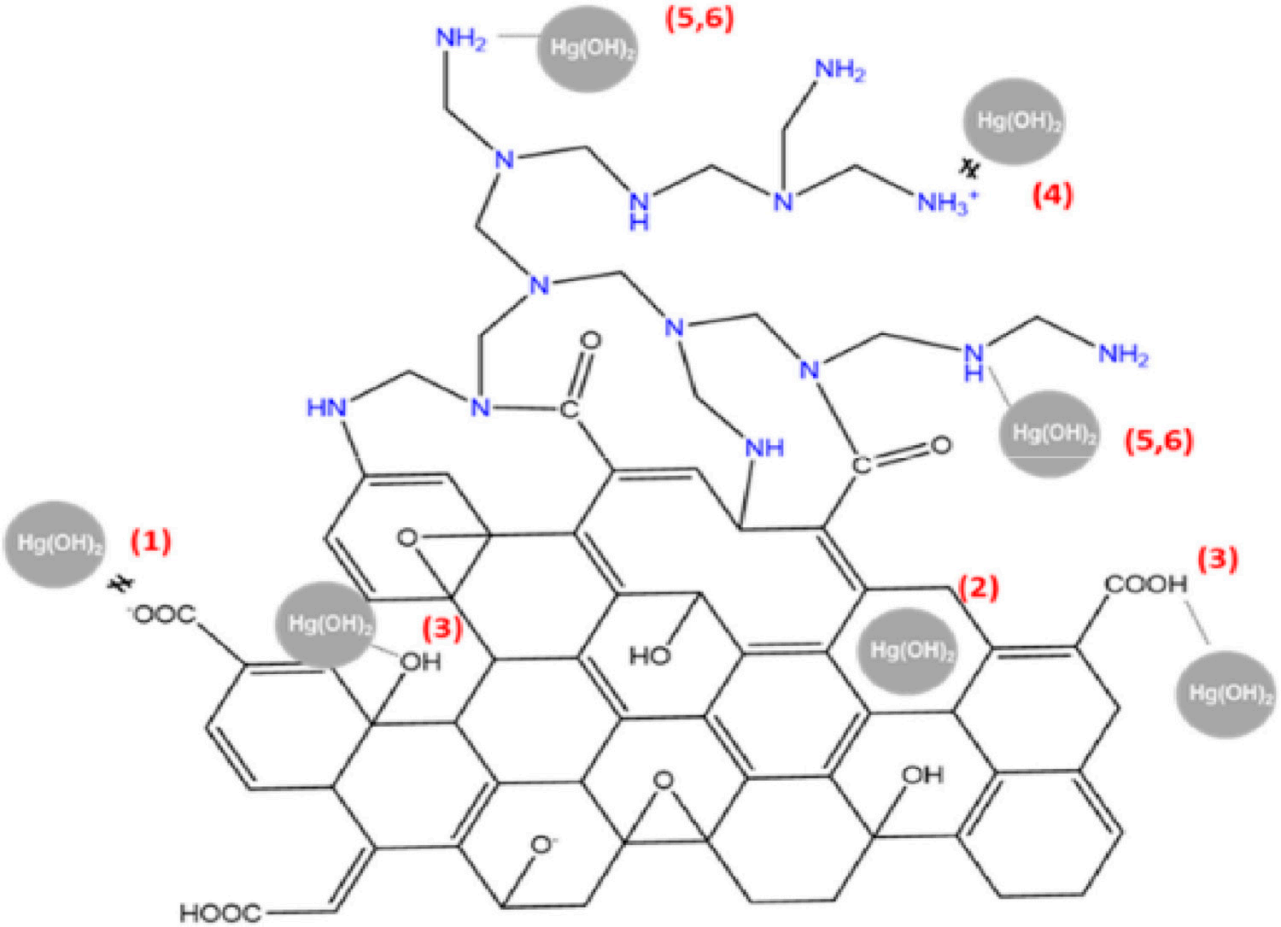
Schematic representation of the potential interactions involved in the removal of Hg (II) (Borrás et al., 2022).

Industrialization has led to a rise in WW discharge. Metals are typical pollutants that may unintentionally contaminate drinking water supplies and aquatic ecosystems as a consequence of anthropological actions, including mining and industrial waste generation, soldered joints, and plumbing material corrosion (Assad and Kumar, 2021; Assad et al., 2023a; Assad and Kumar, 2023; Assad et al., 2023b; Assad et al., 2024). The presence of heavy metals in water can be hazardous to human health as well as negatively impact aquatic life (Yu et al., 2019). Heavy metals do not biodegrade like organic substances. Industrial effluent contains a variety of pollutants, including cobalt, chromium, zinc, lead, mercury, arsenic, and cadmium (Wang et al., 2021). These metals have the potential to be harmful and carcinogenic to living things, even in small amounts (Priyadharshini et al., 2022). Consequently, there is an increasing interest in reducing the amount of hazardous metals in aqueous medium (Zhan et al., 2019). Metals and organic molecules have been eliminated using a variety of physical and chemical techniques, as previously mentioned (Zhu et al., 2010). These techniques, however, have poor removal efficiency. The adsorption process is the most popular approach to treating water as it is inexpensive, simple to use, effective, and environmentally benign. Additionally, adsorbents can be recycled *via* the desorption process (Elsagh et al., 2017). Furthermore, adsorption does not result in the creation of toxic materials (Ahmaruzzaman, 2011). Using GBNs as adsorbents may have several benefits. First, two basal planes in single-layered graphene materials are available for the adsorption of pollutants. By contrast, the adsorbates cannot access the inner walls of CNTs. Second, without the need for complicated machinery or metallic catalysts, GO and rGO can be produced simply through the chemical exfoliation of graphite (Zhao et al., 2014). There are no catalyst remnants in the finished graphene material; therefore, no additional purifying procedures are required. Concerning GO, no extra acid treatments are necessary to give the material a hydrophilic character and reactivity because it already has a significant amount of oxygen-comprising functional moieties. Given that those FGs probably cause metal ions to adhere to GO sheets, this is a huge advantage. Numerous studies have discussed the use of GBNs as adsorbents to confiscate inorganic species from water. For the majority of these investigations, GO was used as a model adsorbent to eliminate metallic ions from H_2_O (Sitko et al., 2013). Because GO has a higher concentration of oxygen groups that can communicate with metallic ions than pristine graphene, GO is preferred for metal ion adsorption. The effectiveness of Pb (II) adsorption on unoxidized and oxidized graphene sheets was compared to highlight the significance of these oxygen-comprising functional units. To incorporate oxygen functional units, pristine graphene was initially processed using a vacuum-promoted low-temperature exfoliation. This was shadowed by heat treatments (GNS500 and GNS700) at 500°C and 700°C. In comparison with pristine graphene, the abovementioned structures displayed a greater capacity for adsorbing divalent lead ions, which emphasizes the significance of carboxyl units in the adsorption procedure of lead ions (Huang et al., 2011). The adsorption efficiency of GO was discovered to be influenced by several variables, including the strength of the ions, pH, GO layer count, and the occurrence of natural organic matter. Recently, Tan et al. (2015) created GO membranes, and they were employed as adsorbents to remove Cu (II), Cd (II), and Ni (II) with the highest adsorption capabilities of 72.6, 83.8, and 62.3 mg/g, respectively. The greater interlayer spacing of the GO membranes allowed the adsorption to achieve an equilibrium state faster (10–15 min), which is advantageous for promoting the interstitial diffusion of HMIs to functional sites. Over six regeneration cycles of the GO membranes resulted in a minor reduction in their adsorption capability (Tan et al., 2015). Zhao and coworkers revealed that at pH 6.0, copper and cadmium divalent ions were successfully adsorbed by GO sheets (Zhao et al., 2011), whereas Sitko et al. verified the elimination of copper, zinc, cadmium, and lead divalent ions at pH 5.0 (Sitko et al., 2013). Yari et al. (2016) employed GO as an adsorbent to remove Pb (II). The equilibrium time of adsorption on the GO surface was 60 min in this experiment. In this study, the adsorption capacity (AC) of Pb (II) on the GO surface developed in proportion to ambient temperature, i.e., when the temperature rose from 288 to 308 K, the AC increased from 15.9 to 19.7 mg/g. The endothermic character of Pb (II) adsorption on GO is demonstrated by this result. Higher temperatures hence promote adsorption. GO provided a ΔH° value of 22.70 (kJ/mol). This suggests that Pb (II) was adsorbed physicaly on the GO surface. The results of a thermodynamic investigation showed that Pb (II) ion adsorption on the surface of GO was endothermic and spontaneous (Yari et al., 2016). Moreover, the pH_pzc_ (pzc, point of zero charge) of GO in aqueous solution controls how it behaves. The GO surface is negatively charged when pH_pzc_ < solution pH due to the proton removal from carboxyl and hydroxyl fragments. The electrostatic interaction with positively charged metal ions is more advantageous when the GO outer layer is negatively charged, improving AC. The most popular method for creating GBNs (composites) for the elimination of metallic ions is the conjugation of graphene with magnetic NPs, such as Fe or iron oxide (Gollavelli et al., 2013). The elimination of anionic contaminants from aqueous systems, like phosphate (PO_4_^−^), perchlorate (ClO_4_^−^), and fluoride (Kamangerpour et al., 2002), has also been examined using GBNs, although the preponderance of reports focus on the adsorption of cationic metallic ions (Shi et al., 2012). The mechanism of anion adsorption, such as that of halide ions, was traditionally attributed to anion interactions rather than the immobilization of cationic metal species. The basis for this anion association is the interaction between the anion (or lone electron pair) and an aromatic framework on the graphene sheet that is electron deficient. Additionally, Alejandro presents findings on the synthesis of an aerogel incorporating reduced graphene oxide (rGO) and polyethylenimine (PEI) using a supercritical CO_2_ method (Borrás et al., 2022). The synthesized rGO/PEI aerogel demonstrates high efficiency as a sorbent for treating Hg (II)-contaminated water. Sorption tests show the rapid removal of Hg (II) from water, achieving residual concentrations as low as 3.5 μg L^−1^ in a short period, nearing the legal limits for drinking water. The aerogel displays a remarkable maximum sorption capacity of approximately 219 mg g^−1^ for Hg (II), making it a promising candidate for treating Hg (II) contaminated wastewater. Based on the analysis of surface charge, Figure 7 schematically illustrates the potential interactions involved in the removal of Hg (II).

Still, there are undoubtedly challenges with the way that heavy metals are now treated in both industrial effluent and point-of-use water. Current methods typically face constraints and difficulties when it comes to naturally removing certain heavy metals from point-of-use water. The heavy metals in industrial effluent are precipitated as sludge that requires additional treatment, which greatly reduces the value of the metals. Using a GO-modified carbon felt (CF/GO) electrode, an electrochemical method has been devised that can handle heavy metal pollution at low and high concentrations. The methodology uses both direct-current (dc) and alternating-current (ac) electrodeposition (ED) (Liu et al., 2019). Because of the high density of surface FGs in GO, which allows for electrodeposition with >29 g of heavy metal per 1 g of GO, the ensuing AC is two orders of magnitude greater than that of traditional adsorption methods. When used on point-of-use water with low levels of heavy-metal contamination, Dc ED with a CF/GO anode may minimize contamination from single HMIs (Cu, Pb, and Cd) and multiple-ion composites to levels considered safe for human consumption. As with conventional adsorption techniques, this approach can handle a broad array of HM pollutants in point-of-use water. The study’s findings indicate that dc ED is capable of recovering over 99.9% of HMIs from industrial WWs that have high levels of pollution (Liu et al., 2019). Furthermore, the ED technique may selectively recover Cu, Pb, and Cd separately by adjusting the voltage and ac frequency; this increases the elimination of HMs. Table 1 provides a comprehensive overview of significant studies investigating the efficacy of GBNs as adsorbents for the removal of heavy metals from water.

**TABLE 1:** Adsorption characteristics of heavy metals by different GBNs.

S. No	Adsorbent	Contaminant	$q_{m}$ (mg/g)	Medium	References
1	RGO/CoFe_2_O_4_	Pb(II)	299	Aqueous solution	Zhang et al. (2014)
2	PEI-modified GO-alginate hydrogel	Hg	374	Wastewater	Arshad et al. (2019)
3	Thiol-functionalized GO/Fe-Mn	Hg	233.17	Surface water, groundwater, and seawater	Huang et al. (2019)
4	GO-based Fe-MgOH	Ag	142.2	Polluted water	Huang et al. (2018)
5	3-Mercapto propyl-trimethoxysilane functionalized MGO	Hg	129.7	Aqueous solution	Mohammadnia et al. (2019)
6	GO	Pb		Polluted water	Madadrang et al. (2012)
7	GO-manganese ferrite	As(V)	102	Water	Shahrin et al. (2018)
8	rGO	Cd	-	Polluted water	Wu et al. (2015)
9	GO-based Fe-Mg (Hydr)oxide	Pb	617.3	Polluted water	Huang et al. (2018)
10	ß-cyclodextrin decorated GO	Pb	149.56	Polluted water	Zheng et al. (2018)
11	RGO/CoFe_2_O_4_	Hg(II)	158	Aqueous solution	Zhang et al. (2014)
12	GO-based Fe-Mg (Hydr)oxide	Cu	432.9	Polluted water	Huang et al. (2018)
13	PEI modified GO-alginate hydrogel	Pb	602	Wastewater	Arshad et al. (2019)
14	N-doped magnetic GO	Co	14.6	Wastewater	Wang et al. (2018)
15	GO-based Fe-Mg (Hydr)oxide	Zn	121.7	Polluted water	Huang et al. (2018)
16	PEI modified GO-alginate hydrogel	Cd	181	Wastewater	Arshad et al. (2019)

### 5.2 Removal of volatile organic compounds

According to the WHO, different types of volatile organic compounds (VOCs) are to blame for the rise in sarcoma rates among individuals worldwide (Fraga et al., 2019). One VOC and the main indoor air contaminant linked to sick building syndrome is formaldehyde, which is found in varnish and other decorating supplies. Recent studies on VOC eradication through the sorption process and PC degradation have used GBNs, particularly GO, to minimize the damage that VOCs cause to humans and the surroundings (Wang et al., 2013). Owing to the oxygen FGs on its outer-layer, GO is probably going to have a lower hydrophobicity than rGO and pristine graphene. As an outcome, GO might have a lower ability for adsorbing aromatic VOCs than the abovementioned analogs. For instance, rGO and GO were revealed to have adsorption capabilities of 276.4 and 216.2 mg g^-1^, respectively, in an ongoing flow reactor with an initial loading of 50 ppm C_6_H_6_. In comparison with GO, rGO can play a major part in boosting the adsorption capability of aromatic VOCs due to its hydrophobic nature and higher inclination to form π–π bonds. In addition to hydrophobicity, it was hypothesized that the superior adsorptive functioning of rGO was a result of its larger surface area compared with GO. It was discovered that rGO and GO had surface areas of 292.6 and 236.4 m^2^ g^-1^, respectively (Yu et al., 2018). Toluene removal tests have also been conducted on the GO and rGO. When demonstrating the desired adsorption of toluene on their outer layers, the ability of GO and rGO to demonstrate π–π bonds, hydrophobic interactions, and electrostatic attraction with toluene can be helpful. Three different forms of GBN-graphene platelets (GP), rGOMW, and KOH were mentioned in a study. Toluene adsorption tests on activated rGOMW (rGOMWKOH) were conducted, and the results obtained contrasted with AC (Kim et al., 2018b) (it is noteworthy that the air conditioner used in this instance is often used as a marketable air conditioner adsorption filter). In removing the toluene, the adsorption capabilities of these GBNs were 2, 7, and 14.4 mg g^-1^ for GP, rGOMW, and rGOMWKOH respectively, and were ordered as follows:

GP <rGOMW <rGOMWKOH

Additionally, it was noted that the composite of GO and MOF-5 was effective at removing benzene gas, with an elimination capability of 251 mg g^-1^ (Liu et al., 2015a). Because there were thought to be weak and non-selective adsorption dynamisms between tiny molecules and MOFs, it was hypothesized that, despite their great porosity, MOFs were unable to hold onto tiny molecules in ambient settings. The aforementioned issues in holding small molecules were resolved by combining graphene-based materials with MOFs. In this context, different amounts of GO, such as 1.75, 3.5, 5.25, and 7 wt%, were used to construct the GO/MOF-5 composite. From all of these composites, the one made with 5.25 weight % GO had the maximum SA and volume of pore among the evaluated GO and MOF-5 ratios, making it the best responder in terms of benzene removal capability. Aliphatic VOCs, particularly n-hexane, are commonly released into the environment (Sun et al., 2014). The industries that typically use n-hexane are those that produce shoes, bags, electronics, foodstuffs, lubricant extraction, and chemicals. Adsorption of n-hexane is typically regarded as a secure, quick, and affordable way of mitigating it. It was discovered that using a GO/MIL-101 composite was a virtuous process to erase n-hexane from the atmosphere. Despite the excellent n-hexane elimination performance of graphene-based composites, few studies have been conducted in this field. RCHO and RCOR are the chief carbonyl VOCs influencing the atmosphere. GBNs have also been successfully employed to eliminate carbonyl VOCs. An amino-functionalized graphene aerogel was employed to remove gaseous formaldehyde in a study, both in its pure usage and as a compound with CNTs (Wu et al., 2017a). Chemical and physical adsorption techniques were used to bind formaldehyde to the aforesaid amino-functionalized graphene sheets. The van der Waals interactions *via* amino and carbonyl moieties of CH_2_O were principally accountable for the chemical adsorption process. CNTs supported the graphene layers in the CNT-adjusted amino-functionalized graphene aerogel (GN/E), which decreased the pore diameter. The adsorptive functioning of GBNs has also been investigated for ketonic VOCs (acetone and butanone) in addition to aldehydes (Zhou et al., 2014; Guo et al., 2016).

Generally, the elimination of various VOCs showed excellent promise when using graphene materials both on their own and in combination with other strong structures. According to several studies, these GBNs are far superior to traditional adsorbents like AC and zeolites. Nonetheless, experimental variables like high or low partial pressures of the target gaseous molecules may drastically change how well the adsorbent material performs. It is noteworthy to mention that some adsorbents function exceptionally well under controlled circumstances. In more real-world settings, in which the dosage of the target contaminant is lower than in an experiment, these NMs, can function very badly. To eliminate the systematic bias in such concerns, it is crucial to evaluate the effectiveness of adsorbents by using appropriate metrics (including the partition coefficient (PC)) to prevent these difficulties (Szulejko et al., 2019).

### 5.3 Removal of antibiotics

**FIGURE 8:**
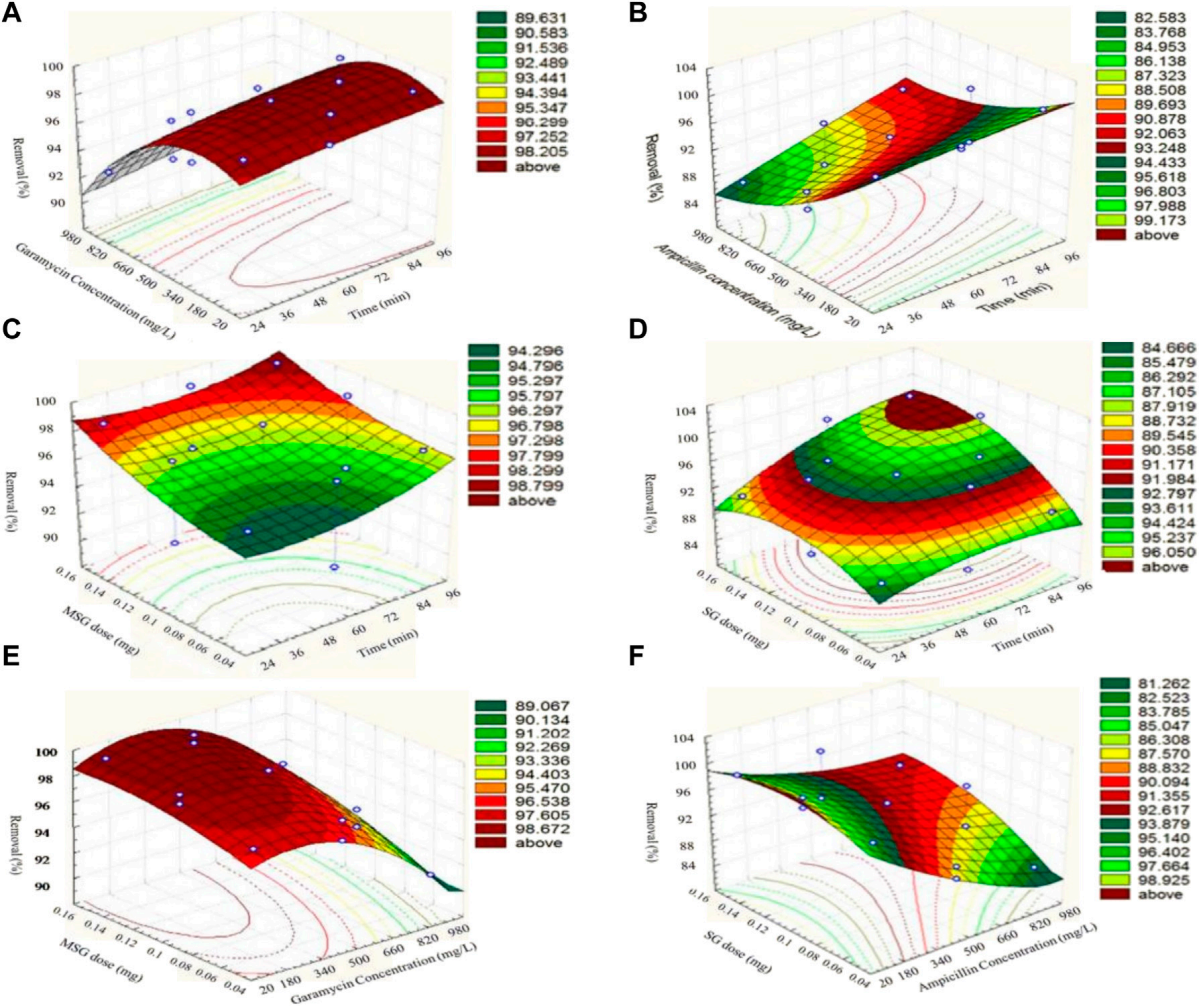
Surface plots representing the removal efficiency (%) of antibiotics (garamycin and ampicillin) on magnetic-functionalized graphene nanocomposites (MSG and SG, respectively) (Elessawy et al., 2020). **(A, C, E)** represent Surface plots of response for removal efficiency (%) of Garamycin on MSG, **(B, D, F)** represent Surface plots of response for removal efficiency (%) of Ampicillin on SG.

Pharmaceutical medications are a class of organic pollutants that detrimentally impact public health and the atmosphere. Between 30% and 90% of these substances are still not degradable and are ejected as active molecules in the surroundings, even at trace levels (Siddiqui and Chaudhry, 2018). The application of GBNs for the adsorption of antibiotics has demonstrated potential in research (Zhao et al., 2020). The adherence of organic materials on the interface of GBNs is thought to be caused by five different possible interactions, comprising hydrophobic effects, π-π- stacking, H-bonds, covalent contacts, and electrostatic relations (Kim et al., 2018a). When used as an adsorbent, graphene has great qualities for removing antibiotics. As graphene is composed of just one C-sheet, all of its atoms are easily contacted by antibiotics due to their exposure to their surroundings on both sides (mostly through a π-π interaction). Second, the porous shape and large surface area of graphene adsorbents, as compared with conventional adsorbents, make them a prime option for antibiotic surface reactions or quicker diffusion, resulting in efficient and rapid adsorption (Perreault et al., 2015). Third, the cost of producing graphene adsorbents on a wide scale is less expensive than that of other high-performance adsorbents (such as carboxyl multi-walled carbon nanotubes and single-walled carbon nanotubes) with an equivalent antibiotic AC. Moreover, antibiotic-polluted water and other organic and inorganic pollutants can be efficaciously remediated using GBNs, as shown in Figure 8.

Despite significant advancements in graphene adsorbent technology, many intrinsic drawbacks remain to be addressed. Surface hydrophobicity and facile agglomeration in aqueous solution are graphene’s primary disadvantages, which significantly reduce the material’s adsorption capability in real-world applications (Bai et al., 2015). Thus, the development of functionalized graphene is required to address these limitations (Kumar et al., 2017). Chemically modified graphene has the potential to generate oxygen-containing groups linked to its carbon backbone, improving its dispersion and resulting in a homogenous aqueous suspension. Two significant GBNP branches created by chemically altering graphene are rGO and GO. Research has used GO and rGO as adsorbents in numerous experiments to retrieve several antibiotics from water-based solutions (Rostamian and Behnejad, 2016). According to Chen et al. (2015), GO efficiently adsorbs SMX and CIP at pH 5.0, with maximum sorption capacities of 240 and 379 mg/g, respectively. Liu et al. looked into the adsorption of two sulfonamide pharmaceuticals from water using two rGOs (Liu et al., 2016). These experimental findings indicate that GO and rGO have a great deal of potential for absorbing various antibiotics.

Owing to the existence of functional moieties (such as ^●^OH and ^●^O2^-^) created by photocatalysts, antibiotics can be successfully degraded or reduced into non-hazardous tiny molecular entities under sunlight, VL (VL), or UV light in addition to adsorption (Li et al., 2016a). Thus, PC degradation is among the most well-liked, productive, and eco-friendly techniques for eliminating environmental contamination caused by antibiotics. Its high electrical conductivity, low manufacturing cost for large-scale manufacturing operations, and specific surface area for even distribution, the quick transmission of ēs, and narrow band-gap energies, have made graphene a potentially lucrative photocatalyst that has been comprehensively explored for the PC breakdown of antibiotic pollutants in water. However, studies have shown that GO cannot function in the VL area because its band gap is just 1.79 eV (Anirudhan et al., 2017), and it easily loses its catalytic properties during the self-aggregate procedure between the layers of graphene (Julkapli and Bagheri, 2015). Consequently, to get around these problems and increase the catalytic activity of antibiotics, graphene is frequently mixed with other photocatalysts to create innovative graphene-based photo-catalysts (GBPs). Various efforts (including the use of different semiconductors) have been made to create and manufacture GBPs in the last few years to increase the degradation capacity of antibiotic pollutants. Antennas based on graphene-based nanosheets are an effective solution for mitigating the many drawbacks of individual semiconductors. Rich surface FGs and a large specific SA of graphene materials may be linked to their strong PC qualities, which help to increase adsorption efficiency. Furthermore, combining different graphene materials may hasten the electron transport and separate photo-induced electron-hole pairs more quickly. The rGO/Bi_2_WO_6_ composites were produced by Anirudhan et al. (2017) and used to remove CIP in a VL simulation. The rGO/Bi_2_WO_6_ composites demonstrated an exceptional VL-driven PC degradation rate of CIP (89.2%). This was most likely caused by the rGO loading, which decreased the rate of electron-hole recombination while simultaneously increasing adsorption and catalytic sites. The produced ē could be effectively transferred from the CB of Bi_2_WO_6_ to rGO under VL irradiation, prolonging the lifespan of photo-excited ē/h^+^ couples. This portion of the electrons could come into touch with the O_2_ present in the photodegradation system at the same time and react swiftly to create ^●^O_2_^−^ groups. The ^●^O_2_^−^ and h^+^ produced by photolysis can react further in water to break down CIP molecules. This study demonstrated that adding Bi_2_WO_6_ to rGO as a photocatalyst could effectively increase the PC activity of CIP degradation under VL by ensuring higher electronic conductivity, accelerating the separation of photo-induced electron-hole pairs, and prolonging the electron transfer. Currently, an increasing number of studies have concentrated on linked semiconductor materials in conjunction with graphene, which typically offers the major benefits of encouraging electron-hole pair separation and preserving the reduction and oxidation events at two distinct reaction sites (Tang et al., 2015). For example, the coupling of other semiconductors (ZnS, ZnO, *etc.*) with CdS-graphene composites has garnered a lot of interest because of its benefits, which include increasing charge separation, extending the life of the charge carrier, and increasing the effectiveness of charge transmission (Huo et al., 2016). Furthermore, an Ag_3_PO_4_/BiVO_4_/rGO heterojunction photocatalyst was successfully prepared by Chen et al. (2017) using a simple *in situ* deposition method. The catalyst demonstrated 90% removal effectiveness toward TC under VL irradiation, which was significantly higher than that of pure BiVO_4_ (56%), Ag_3_PO_4_/BiVO_4_ (82%), and rGO/BiVO_4_ (78%). Compared with single semiconductors or single semi-conductor/graphene composites, the PC activity of coupled semiconductor/graphene composites can be significantly increased. Therefore, creating a novel coupled semiconductor/graphene multi-component photocatalyst and investigating the processes of component interaction are crucial for the removal of antibiotics.

Additionally, by combining the benefits of graphene structure with these materials, other methods such as combinations with other functional nanoparticles, polymers, or optical fibers can also operate as an extremely effective composite substance for eliminating various antibiotics (Cao et al., 2016; Anirudhan and Deepa, 2017; Lin et al., 2017b). These substances could substantially improve the photocatalytic effectiveness for antibiotic elimination by improving the surface area, responsiveness, stability, active sites, charge separation and transfer, and reaction of the photocatalyst. Lin et al. created TiO_2_-rGO to eradicate SMX pollution in water using an immobilized photoreactor and catalyst-coated side-glowing optical fibers (SOFs). Higher light utilization efficiency was achieved with SOFs by promoting light transmission directly through the inner fiber cores to the photocatalysts coated on the surface and the outer surface of the photocatalysts. This was a cost-effective way to transfer photons in big reactors evenly and efficiently without having to separate the photocatalysts from the water (Lin et al., 2017a). Table 2 provides a comprehensive overview of significant studies investigating the efficacy of GBNs as adsorbents for the removal of antibiotics from water.

**TABLE 2:** Adsorption characteristics of antibiotics by different GBNs.

S. No	Adsorbent	Contaminant	$q_{m}$ (mg/g)	Medium	References
**1**	GO	SMX	240	Polluted water	Chen et al. (2015)
**2**	rGO	SMZ	174.42	Aqueous solution	Song et al. (2016)
**3**	GO nanosheet	SMX	127	Water	Rostamian and Behnejad (2016)
**4**	Graphene nanosheet	SMX	103	Water	Rostamian and Behnejad (2016)
**5**	Fe/Cu-GO	TC	201.9	Water	Tabrizian et al. (2019)
**6**	Fe_3_O_4_@G	TC	423	Water	Zhang et al. (2017)
**7**	GO/TiO_2_	CTC	261.10	Aqueous solution	Li et al. (2017b)
**8**	M-GNPs	AA	106.38	Aqueous solution	Kerkez-Kuyumcu et al. (2016)
**9**	Nano-GO/M	CIP	1.36	Polluted water	Alicanoglu and Sponza (2017)
**10**	MGB	TC	388.33	Aqueous solution	Li et al. (2017a)

Although graphene-based photocatalysts can photodegrade antibiotics, there is currently very little research regarding the application of graphene-based photocatalysts for antibiotic removal and the photodegradation of antibiotics in combination with other materials. Thus, it is crucial to investigate the possibilities of these materials in photocatalysis.

### 5.4 Persistent organic compounds

**FIGURE 9:**
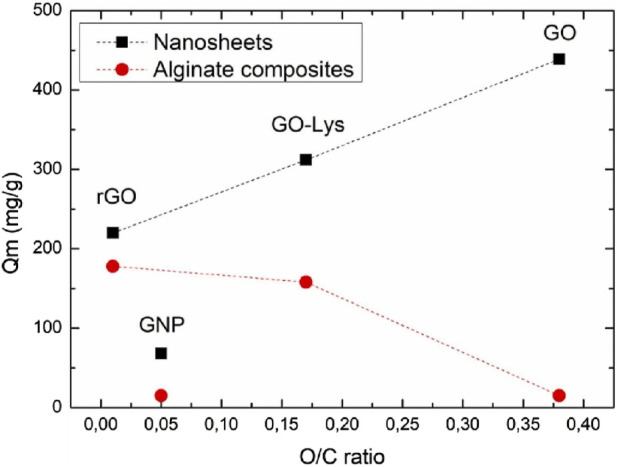
Monolayer adsorption capacity ( $Q_{m}$ ) of RhB as a function of the oxidation degrees expressed as the O/C ratio (Tunioli et al., 2023).

Persistent organic compounds (POCs) constitute a class of organic chemicals characterized by their resistance to environmental degradation, stemming from their chemical stability and resistance to breakdown processes (Radjenovic and Sedlak, 2015; Mantovani et al., 2023). Examples include polychlorinated biphenyls (PCBs), dioxins, furans, organochlorine pesticides, poly-brominated diphenyl ethers (PBDEs), and per- and poly-fluoro-alkyl substances (PFAS) (Schulze et al., 2019; Gagliano et al., 2020; Podder et al., 2021). POCs, which are prevalent due to past industrial use, waste incineration, and certain consumer products, pose environmental and human health risks. Their persistence in ecosystems, ability to bio-accumulate, and potential long-range transport underscore the need for global regulatory efforts to mitigate their impact on environmental integrity and public health. Graphene’s unique properties (Khaliha et al., 2021; Mantovani et al., 2021; Lombardi et al., 2022) make it an ideal candidate for adsorption and catalysis in the removal of POCs from water. Graphene oxide (GO) and reduced graphene oxide (rGO), which are derivatives of graphene, exhibit increased adsorption capabilities due to their functional groups and large specific surface area (Mantovani et al., 2022; Mantovani et al., 2023). These nanomaterials can effectively capture POCs through π-π stacking, hydrogen bonding, and other interactions. Additionally, the incorporation of other nanoparticles onto graphene surfaces can further increase their adsorption capacities. The efficient regeneration of graphene-based adsorbents adds to their appeal for long-term use. Thus, the utilization of graphene-based nanomaterials for the removal of POCs has emerged as a promising avenue in environmental remediation. For instance, Ren et al. investigated the adsorption behavior of six polychlorinated biphenyl (PCB) congeners using pristine graphene (GN), graphene oxide (GO), and sulfonated graphene (SG) at environmentally relevant concentrations ranging from picograms to micrograms per liter (Ren et al., 2019). GN demonstrated superior adsorption capacities to GO and SG, which was primarily attributed to increased surface adsorption. A conspicuous planarity effect was observed in the adsorption of PCB congeners on all graphene nanomaterials at low aqueous concentrations (0.01–10 ng L^−1^), which diminished as the concentration increased. Notably, functionalized graphene, especially SG, exhibited a more pronounced planarity effect than pristine GN, particularly at lower concentrations. Under acidic or alkaline conditions, the planarity effect on GO was attenuated due to the preferential dispersion of GO particles in the solution. However, the planarity effect on SG remained minimally impacted by changes in the pH of the solution. Lower temperatures increased the planarity effect in the adsorption of PCBs on both functionalized graphene materials, with SG displaying a lower increase than GO. Conversely, elevated temperatures resulted in the suppression of the planarity effect. Additionally, a boron-doped graphene sponge anode was synthesized and employed by Nick et al., for the electrochemical oxidation of C4-C8 per- and poly-fluoroalkyl substances (PFASs) (Duinslaeger and Radjenovic, 2022). Operating in low conductivity electrolyte and one-pass flow-through mode, removal efficiencies ranged from 16.7% to 67% at an anodic current density of 230 A m^−2^, with an energy consumption of 10.1 ± 0.7 kWh m^−3^. The removal mechanisms included electrosorption (ES) (7.4%–35%) and electro-oxidation (9.3%–32%). Defluorination efficiencies for C4-C8 per-fluoro-alkyl sulfonates and acids ranged from 8% to 24%, with efficient fluoride recovery (74%–87%) suggesting effective C-F bond cleavage. Stoichiometric sulfate recoveries (91%–98%) indicated the proficient cleavage of sulfonate head-groups. Adsorbable organic fluoride analysis revealed the ES of remaining partially defluorinated byproducts during the current application, which were released into the solution after current cessation. This proof-of-concept study highlights the graphene sponge anode’s capability for C-F bond cleavage and defluorination of PFAS, offering the potential for the electrochemical degradation of PFAS-laden wastewaters and brines due to the anode’s electrochemical inertness toward chloride and absence of chlorate and perchlorate formation, even in brackish solutions. Moreover, graphene nanosheets and nanoplatelet-alginate composite hydrogels were synthesized by Francesca et al., through ionic gelation for the removal of emerging contaminants (ECs) from tap water (Tunioli et al., 2023). The resulting gel beads exhibited a porous structure with a uniform distribution of graphene materials on pore surfaces. The adsorption kinetics of graphene-related materials (GRMs) were notably faster than granular activated carbon (GAC), a benchmark industrial sorbent, with an ofloxacin removal capacity 2.9 to 4.3 times higher. Confocal Raman microscopy mapping and SEM confirmed the gel bead structure and GRM distribution. Adsorption isotherm studies, as shown in Figure 9, revealed a high maximum adsorption capacity of 178 mg/g for rhodamine B, which was comparable with powered activated carbon.

**FIGURE 10:**
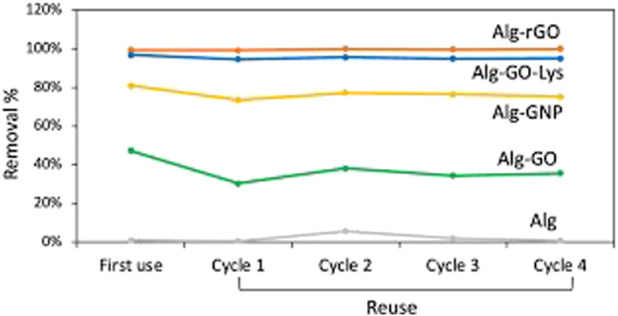
Regeneration test of alginate and alginate-graphene beads (Tunioli et al., 2023).

Regeneration tests, as shown in Figure 10, demonstrated the resilience of the gel beads, maintaining adsorption performance even after saturation and washing with ethanol. Repeated reuse cycles up to the fourth cycle showed no significant loss of adsorption efficiency. These findings highlight the potential of graphene-based composite hydrogels for effective EC removal, including bisphenol A, ofloxacin, and diclofenac, from tap water. The study suggests the promising application of these materials in water treatment, offering advantages in terms of both adsorption kinetics and recyclability.

Overall, the application of graphene-based nanomaterials in POC removal showcases a promising and sustainable approach to addressing environmental pollution challenges. Ongoing research continues to explore and optimize these materials for practical and large-scale remediation applications.

### 5.5 Other contaminants

Surface area and pore size distribution are the main surface characteristics that affect adsorption on graphene. Although there is no porosity, pristine graphene possesses an extremely high specific surface area. Porosity can be added to graphene to significantly increase its adsorption efficacy by mixing it with other porous materials, such as silica, chitosan, and gelatin (Lin et al., 2017a; Fan et al., 2019). By adding various functional groups, good adsorption performance can also be attained. The formation of bonds with the adsorbate is facilitated by these functional groups. The example of GO and rGO, which have many oxygen-containing functions that can form bonds with the adsorbate, makes this quite visible. Consequently, the successful application of functionalized graphene-based nanocomposites in environmental remediation depends on their ability to be fabricated with a wide surface area, high porosity, and oxygen-containing functionalities, as demonstrated in section 4. As this section discusses, graphene-based adsorbents have been used to remove a variety of organic contaminants thus far, but with many changes made to the original graphene structure.

Using graphene-based materials to remove aromatic polycyclic polar and non-polar chemicals has been reported in multiple cases. The adsorptive removal of polar and non-polar PAHS is facilitated by the π-π interactions, hydrophobic effect, and van der Waals forces between graphene-based adsorbents and PAHS. Colloidal graphene oxide nanoparticles (GONPs) were used to study the adsorptive characteristics of a variety of aromatic compounds, including polar (1-naphthylamine and 1-naphthol) and non-polar (PYR, PNT, NAP, and DCB) molecules. This research was conducted by Wang et al. (2014). The strong hydrophobicity of PYR and PN was the cause of their high adsorption affinities. The adsorption affinity order for the hydrophobic effect was determined by normalizing the adsorption data, and it was PYR > PNT > NAP > DCB. In addition to the hydrophobic impact and van der Waals forces, π electron-rich PAHs and electron-depleted portions of strongly polarized graphitic surfaces communicate through π-π electron donor-acceptor (EDA^+^) interactions. The adsorption capacities of GONPs were increased by the π-π interactions, Lewis acid-base interactions, and H-bonding between 1-naphthylamine and 1-naphthol (Wang et al., 2014).

Just as there are several papers on PAHs, graphene-based adsorbents have also shown promise for phenolic compound removal. In-depth adsorption is influenced by the adsorbent surface area and degree of reduction. According to Wang et al. (2015), PNT and 1-naphthol significantly increase the adsorption affinities of GO nanoparticles (GONPs) in the presence of sulfide. The increased surface hydrophobicity of GO following Na_2_S treatment was the cause of the higher PNT adsorption. The increase in adsorption in the naphthol example resulted from the phenolic and carboxyl groups on the surface of GO converting from epoxy/ether groups, allowing for a deeper H-bonding between 1-naphthol and GONPs. Another study examined the theoretical and experimental relationships between phenol naphthol and rGO (Yu et al., 2017). Both phenol and naphthol were adsorbed on rGO, according to the pseudo-first-order model. When the pKa value was reached, the removal capacity increased along with the pH, at which point the negatively charged rGO and anionic phenols experienced electrostatic repulsions. The π-π EDA interactions caused the phenols to be adsorbed. Using the projector augmented wave (PAW) approach and the PBE functional at the GGA, the authors performed DFT. The computed Eads further supported the final closest interaction’s finding that naphthol exhibited stronger π-π interactions with the rGO plane than phenol. The adsorption of 4-CP on pure graphene (Gupta et al., 2018), GO, and N- and B-doped graphene was investigated using DFT (Cortés Arriagada et al., 2013). Because of hydrogen bonding, the 4-CP adsorption on GO was stronger than on PG, as demonstrated by a molecular dynamics simulation using the PM6 potential. Because graphene is an acceptor, the charge density distribution showed electron transport from 4-CP to graphene. However, in the case of GO, it was discovered that the dispersion and H-bonding interactions caused the oxygen functions to take an electron from graphene and then transfer it to 4-CP. The adsorption of N- and B-doped graphene was determined by the authors to be equivalent to that of PG for 4-CP. By raising the doping concentration on the surface, it is possible to somewhat increase the adsorption on N-doped graphene. Using DFT-D3 computations with the revPBE/def2-TZVP theory model, Abadee et al. (2019) recently investigated the interaction of phenol and water molecules with graphene and graphene nanobuds. As these forces have an impact on the binding behavior of the interacting species, the DFT-D3 research investigation of non-local dispersion forces is crucial in this situation. In comparison to water molecules, greater phenol adsorption was made possible by the simultaneous presence of π-π stacking and electrostatic interactions on graphene and graphene nanobuds. Because of its higher SSA, graphene nanobuds turned out to be a superior adsorbent to graphene. Another theoretical study used DFT research with GO as the adsorbent to examine the adsorption behavior and mechanism of phenol, 4-CP, 2,4-diCP, and 2,4,6-triCP (Wei et al., 2019). They used DFT-D3 simulations to account for dispersion corrections to determine the most stable geometry because the weak interactions in the sorption system have an impact on the geometry. Theoretical data led the authors to conclude that the adsorption process is primarily driven by the hydrophobic effect, H-bonding, and π-π interactions. MD simulations provided more support for this finding. Additionally, it was discovered that adsorption affinity increased with increasing numbers of hydroxyl groups on GO and decreasing numbers of chloro groups on phenols. Moreover, the presence of polar solvents and acidic environments strengthened H-bonding and electrostatic interactions.

Another significant class of organic contaminants is dyes. Dyes are released into the water by a wide range of industries, including printing, textile, dyeing, paper manufacture, tanning, and painting. The majority of colors dissolve in water and are either cationic or anionic. Most dyes have complex chemical structures, are long-lasting, and do not break down naturally. Additionally, some colors are toxic to humans. They disrupt natural cycles and present several health risks to living things (US EPA). For instance, it has been found that approximately 10% of the dyes, which are highly carcinogenic and toxic, are released into WW (Uddin et al., 2009). Furthermore, dyes change the color of water, interfering with aquatic plants' ability to photosynthesize, blocking sunlight, and creating an unbalanced aquatic ecosystem (Thakur and Kandasubramanian, 2019). Therefore, taking into account the possibility of environmental toxicity and public health harm, these dyes must be removed. Van der Waals forces, π interactions, and oxygen-containing groups cause the positively charged amino groups of dye molecules to engage electrostatically with the negatively charged surface of the adsorbate, which is how most dye removals are accomplished.

GO-hydrogel porous nanocomposites were created by Pourjavadi et al. (2016), who also investigated the impact of the hydrogel’s porosity on dye adsorption. By incorporating CaCO_3_ in varying concentrations and then removing it, they were able to get varied porosities. The exceedingly high porosities they discovered allowed for an exceptionally high AC. The Langmuir isotherm model (LIM) and the pseudo-second-order kinetics model provided the best description of the adsorption. To adsorb MB, Mercante et al. (2017) fabricated PMMA nanofibers wrapped with rGO (PMMA-rGO) and found that the spontaneous adsorption was driven by the π-π stacking interactions. The adsorption of MB dye was best characterized by the pseudo-second-order kinetics model and the LIM. Furthermore, Huong et al. (2018) produced magnetic manganese ferrite/GO nanocomposites to adsorb MB dye. According to their proposal, GO nanosheets are primarily involved in the interactions that cause dye molecules to adsorb, such as π-π interactions, oxygen-containing groups, and electrostatic/ionic interactions. To improve electrostatic/ionic interactions, π-π electron coupling, and other oxygen-containing functional groups, such as carboxyl, epoxy, and hydroxyl groups, they raised the concentration of GO, which in turn boosted adsorption activity. The best agreement was found between the adsorption isotherm data and kinetics and the pseudo-second order kinetics model and Langmuir isotherm model. In a different study (Wang, 2017), it was shown that magnetic graphene nanoparticles (Fe_3_O_4_@GNs) had increased MB adsorption capability upon a decrease in C=O groups. The kinetics results demonstrated that MB dye and Fe_3_O_4_@GNs undergo chemisorption through π-π interactions, as predicted by the pseudo-second-order model.

In addition to the cationic dyes previously described, graphene-based adsorbents have been widely employed in the elimination of diverse anionic dyes. GO was used by Konicki et al. (2017) to adsorb the anionic azo dyes acid orange 8 (AO8) and direct red 23 (DR23). The isotherm model approximated the Langmuir form, while the kinetics model neared pseudo-second-order kinetics. Owing to the deprotonation of the -COOH and -OH groups at basic pH, which created electrostatic repulsive forces with the sulfonate anions (RSO_3_^−^) in the dye molecules, the effectiveness of adsorption reduced with increasing pH. Apart from the electrostatic interactions, the adsorption of dyes onto GO was also facilitated by H-bonding and π-π stacking interactions. They proposed that for the DR23 ions to reach the adsorption sites, they must first (at least partially) exit the hydration shell, which necessitates energy input. As a result, for DR23 and AO8, the adsorption process is exothermic and endothermic, respectively. The two color adsorption values for ∆Gº fell within the physisorption range.

The literature makes it abundantly evident that while GO’s anionic groups and anionic dyes experience high electrostatic repulsion, GO shows significant cationic dye adsorption through the formation of electrostatic interactions. Because of the additional stacking interactions, GBNs can effectively behave as excellent adsorbents for cationic and anionic dyes. Wang et al. investigated the adsorption of a neutral dye, acridine orange (AO), in addition to cationic and anionic dyes (Wang et al., 2016). They coated a graphene oxide sheet (GO) with calcium silicate after depositing Fe_3_O_4_ nanoparticles, creating an MGSi graphene oxide composite. Because MGSi contains carboxylic groups, which give its surface a negative charge at pH values greater than 2.8, which are electrostatically attracted toward the positively charged AO, these nanocomposites could adsorb up to 193.05 mg g^-1^. The highest AC was attained at pH 6, and it increased as pH rose. Because AO is neutral and MGSi has a negative charge in the basic medium, the AC is reduced.

Pesticide removal is also a big concern because of the widespread use and careless application of these chemicals in drinking water. Agricultural, dairy, and insect control still use a lot of pesticides, which are organic aromatic compounds. Furthermore, herbicides have been used in veterinary treatment and home gardening. Even in extremely low concentrations, they are dangerous to living things. Because pesticides can cause neurotoxicity and cancer, and are involved in other illnesses, their routine usage is not recommended (Paramasivan et al., 2019). Moreover, acetylcholinesterase enzyme inhibitors, which cause nervous system dysfunction, are the reason that organophosphorus insecticides are hazardous (Fraga et al., 2019). Numerous research teams have also investigated employing graphene-based materials as adsorbents to remove pesticides from aqueous solutions. Strong electrostatic and π-π interactions reduce pesticide absorption. Adsorption efficiencies for ametryn, prometryn, simazine, simetone, and attrazine at pH 5°C and 25°C were reported by Boruah et al. (2017) as 93.61, 91.34, 88.58, 81.22, and 75.24%, respectively, on the Fe_3_O_4_/rGO nanocomposite. Because of the greatest electrostatic interactions and the least amount of iron leaching from the adsorbate, the AC reduced as pH increased and reached its maximum at pH 5. It was discovered that the Fe_3_O_4_/rGO nanocomposite’s oxygen-containing functions and the pesticide’s ≡N^+^‒ group interacted electrostatically. The most beneficial adsorption was facilitated by the strong π-π interactions. Additionally, metal-organic framework/graphene oxide hybrid nanocomposites (UiO-67/GO) were created by Yang et al. (2017a) to remove OPP hydrocarbons. According to the findings, 482.69 mg g^-1^ of glyphosate adsorbed at pH 4 corresponds to the Langmuir model and the pseudo-second-order kinetics model. The XPS tests demonstrated that the favorable adsorption is caused by the interaction between the phosphate group of glyphosate and the oxygen-containing FGs on the surface of UiO-67/GO. The significant increase in crude oil discoveries and the boom in petrol products have had detrimental effects on, and ultimately destroyed, many ecosystems. Furthermore, the main environmental issue that frequently arises in water or along beaches is the seepage of oil from oil drilling facilities. To lessen the negative impact on the marine ecosystem, research on the adsorption of leaky lubricants from contaminated H_2_O has been essential (Tsang et al., 2017). Successful recent studies on the adhesion of oil suspensions on GBNs have demonstrated strong adsorption capacities. Extremely spongy GBNs, or xerogels, have recently been created as state-of-the-art oil adsorbents; several of them include magnetic metallic nanospheres that are connected to them and characterized by high recyclability. Table 3 provides a comprehensive overview of significant studies investigating the efficacy of GBNs as adsorbents for the removal of antibiotics from water.

**TABLE 3:** Adsorption characteristics of different contaminants by different GBNs.

S. No	Adsorbent	Contaminant	$q_{m}$ (mg/g)	Medium	References
1	rGO/TNT	MB	26.3	Wastewater	Nguyen and Juang (2019)
2	GO/MOF-5	Ethanol	158.2	Polluted water	Liu et al. (2015a)
3	Fe_3_O_4_/rGO	Simazine	88.58	Aqueous medium	Boruah et al. (2017)
4	Fe_3_O_4_@GO	MB	131.1	Wastewater	Ganesan et al. (2018)
5	Mg(OH)_2_-GO	CR	118.4	Polluted water	Liu et al. (2015b)
6	HCSs/GAs	MO	344.1	Wastewater	Hou et al. (2019)
7	GA	RhB	111	Contaminated water	Tang et al. (2019)
8	Fe_3_O_4_@SiO_2_/GO	CVL	769.23	Aqueous solution	Pourjavadi et al. (2016)
9	rGO/ZIF-67	CVL	1714.2	Polluted water	Yang et al. (2018b)
10	GO/silk fibroin	MB	1,322.71	Dyeing wastewater	Wang et al. (2019)
11	Fe_3_O_4_/rGO	Prometryn	91.34%	Aqueous medium	Boruah et al. (2017)
12	Cu-BTC@GO	Toluene	838.5	Polluted water	Li et al. (2016b)
13	GO	DR23	15.3	Aqueous solution	Konicki et al. (2017)
14	GA	MB	76.0	Contaminated water	Tang et al. (2019)
15	3D graphene	MO	27.932	Aqueous solution	Labiadh and Kamali (2019)
16	Fe_3_O_4_/rGO	Simetone	81.22%	Aqueous medium	Boruah et al. (2017)
17	GA	MG	352	Contaminated water	Tang et al. (2019)
18	HCSs/GAs	RhB	441.5	Wastewater	Hou et al. (2019)
19	SCGOM	MG	289.1	Aqueous solution	Gao et al. (2015)
20	GO	LEV	256.6	Polluted water	Dong et al. (2016)
21	Fe_3_O_4_/rGO	Attrazine	75.24%	Aqueous medium	Boruah et al. (2017)
22	rGO/NMA	CR	473.93	Single and binary water	Wu et al. (2017b)
23	MF-GO	MB	190.8	Aqueous solution	Huong et al. (2018)
24	rGO	Benzene	276.4	Polluted water	Yu et al. (2018)
25	UiO-67/GO	OPP	482.69	Contaminated water	Yang et al. (2017a)
26	Fe_3_O_4_/rGO	Ametryn	93.61%	Aqueous medium	Boruah et al. (2017)
27	GA	MO	16	Contaminated water	Tang et al. (2019)

## 6 Risks of GBNs to human health and environment

**FIGURE 11:**
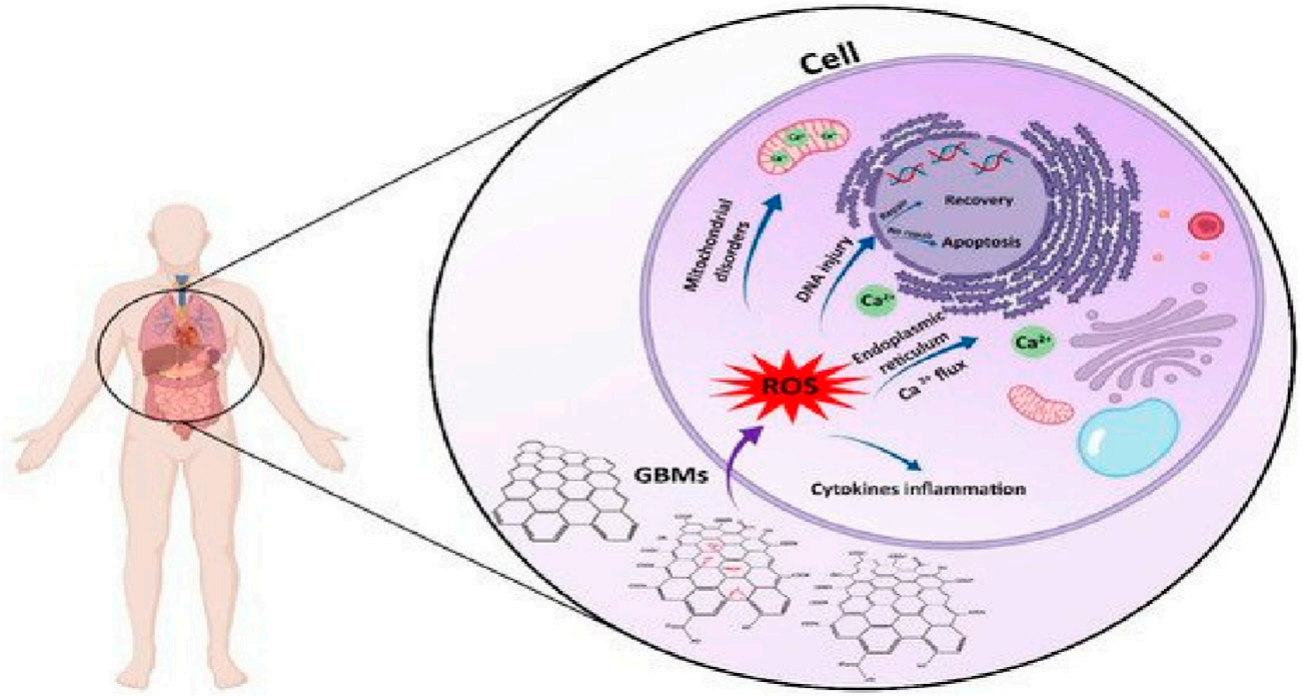
Cellular toxicity of graphene oxide-based NMs (Zare et al., 2021).

NMs based on graphene have been applied in a variety of fields, including biomedicine and environmental exposures. However, even so, the level of toxicity needs to be taken into consideration if it is to be used for human and environmental applications. Understanding and categorizing GBNs according to their applications and safety requirements requires an understanding of how biological characteristics interact with them. When used in non-biomedical applications, GBNs have the potential to be harmful when exposed to the environment (Fadeel et al., 2018). Intentionally or accidently, humans come into touch with GBNs, especially those who create and live in the suburbs of an industrial manufacturing setting. GBNs, which are mostly introduced into the ecosystem through waste from industrial or pharmaceutical production, are exposed by regular people. Different organs may be impacted depending on how GBNs enter the body through biological barriers or blood flow. Owing to their nanosize, surface morphology, complexation, charge, impurities, aggregation, corona effects, and physical destructions, GBNs can pass through the blood-air, blood-brain, blood-testis, and blood-placental barriers, as well as the blood-air, blood, and placental barriers. Several cellular processes, including oxidative stress, DNA destruction, inflammatory response, localized cell death, autophagy, and necrosis, are important contributors to the toxicity of GBNs (Ou et al., 2016), as shown in Figure 11.

**FIGURE 12:**
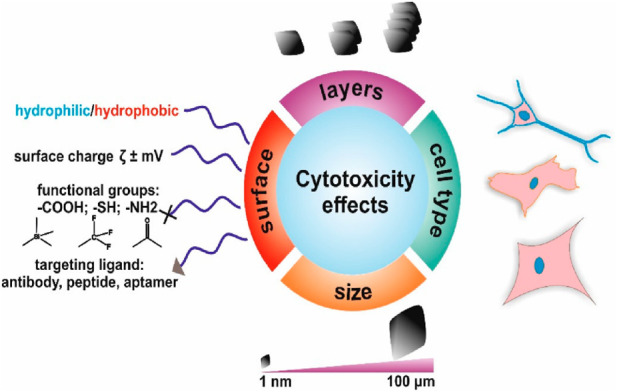
Numerous factors affecting the cytotoxicity of graphene-based materials (Tadyszak et al., 2018).

They represent a significant hazard to the aquatic ecosystem. As a result, GBNs found in soil or water are also ingested by people through a variety of methods, including through the food chain. The environmental effects of GBNs have only been briefly examined by a few researchers (Chowdhury et al., 2015). The immediate impacts of GO on the microbial population in wastewater were examined by Ahmed and Rodrigues (2013). According to their findings, GO was hazardous to microorganisms at concentrations ranging from 50 to 300 mg L^-1^. Because of the increase in water turbidity and decrease in sludge dewaterability, the effluent’s quality worsened. The harmful effects of GO on microbial populations were also confirmed to be caused by the creation of reactive oxygen species (ROS). Hence, it is important to comprehend how GBNs interact with one another and how harmful they are at the cellular and molecular levels. The dose, the form and chemistry of the surface, the exposure route, purity, *etc.*, are some of the elements that are crucial in figuring out the level of toxicity, as shown in Figure 12.

However, Deng et al. looked at characterization elements, including the hazardous impact, destiny, and exposure factor of GO, in the atmosphere to examine the life cycle influence of GO-BNs (Deng et al., 2017). Moreover, the layered architecture of GBNs is controlled by the surface chemistry, which also makes it simpler to comprehend the active surface area and flexural rigidity while carrying out any biofunctionalization feature. The lateral dimension, which is useful for biological aspects including cell uptake, renal clearance, and transit across the blood-brain barrier, can be utilized to ascertain the dimension of the substance. Regarding the use of NMs based on graphene, purity is another issue. The residual and unreacted compounds that are produced throughout the synthesis process must be kept under observation and eliminated. To report the toxicity features of GBNs for biological investigations, numerous characterizations must be carried out. Researchers have suggested using GBNs for a broad array of biomedical purposes by assessing their toxicity and safety in many investigations.

## 7 Conclusion and outlook

The understanding of utilizing graphene-based nanomaterials in various applications, particularly for addressing environmental challenges, has significantly progressed in recent years. The special characteristics of graphene have created new opportunities for augmenting GBN functioning in a broad array of fields, such as wastewater treatment. However, the advancement made possible by the use of graphene was only marginally better than that made possible by other carbon-based NMs or even by more conventional carbonaceous materials, such as activated carbon. In this review, we examined a few current progressions in the fabrication and use of graphene and GBNs in the elimination of contaminants from water. The extraordinary characteristics of GBNs, such as their large SA, several unsaturated π-bonds, mechanical characteristics, and adsorption capacities, have also been elucidated, with a special emphasis on those that favor sensor platforms and environmental applications of this material, such as water remediation. Nevertheless, the utilization of GBNs as adsorbents in the environment should not be limited to water treatment and should to be expanded to include air and soil filtration. Without causing additional deterioration, the high AC and physisorption can be used to remove and separate contaminants from the soil, air, and water. Traditional contaminants including dyes, insecticides, and organic solvents, have been the subject of the majority of investigations. Therefore, research on new contaminants such as oil, grease, antibiotics, phenolics, oxygen-demanding wastes, and derivatives of octanoic acid will be needed in the future.

Despite the numerous indications that demonstrate the value of GBNs as adsorbents, there is no recognition of their extensive use in environmental cleaning. This is because toxicity, which includes both short- and long-term exposure to individuals and their surroundings, is a problem that is almost completely disregarded. As a result, concerns about the impacts on public health and the environment have been raised in the scientific community. As the waste from GBN manufacture is released rapidly into the biological environment, it was expected and later observed that the marketplace for GBN merchandise would reach millions of dollars in coming years. It is essential to have a thorough understanding of the interaction between GBNs and the biological system as well as the possible toxicity of GBNs to the natural environment to fully exploit their application advantages in biomedicine and minimize their influence on the environment. It is obvious that the chemical manufacture of GBNs and their sensor applications is far from mature, and none of them have yet touched the industrial scale, given the quick display of more intriguing features of these materials. Overall, the discussion makes clear that the progress accomplished thus far is impressive. A cost-effective and practical methodology for fabricating high-quality graphene on a wide gage that is also ecologically benign is still needed. In addition, even though numerous studies have demonstrated that the adsorbents of GBNs can be reprocessed, these studies are yet rare, and future studies must focus on more creative approaches to facilitate the parting and renaissance of GBN’s adsorbents.

Furthermore, research on GBNs is still in its early stages and needs additional inputs, but thus far, they seem to be excellent prospects for water treatment applications. The use of GBNs as adsorbents will be revolutionized by more advancements in several fields. Furthermore, with advancements in nanomaterial fabrication, additional graphene-based materials should be produced in future research. Understanding of the many characteristics and phenomena linked to GBNs, especially as adsorbents for water treatment, can be aided by this review, which can also assist researchers in realizing the full potential of GBNs.

## Abbreviations

2D, two dimensional; CBMs, carbon-based materials; CNTs, carbon nanotubes; CVD, chemical vapor deposition; DSSCs, dye-sensitized solar cells; ECL, electrogenerated chemiluminescence; FLG, few-layer graphene; GBNs, graphene-based nanomaterials; GNRs, graphene nanoribbons; GO, graphene oxide; GPs, graphene platelets; GQD, graphene quantum dots; HC, hydrocarbon; LI-rGO, laser-induced reduced graphene oxide; PC, partition coefficient; PZC, point of zero charge; rGO, reduced graphene oxide; SA, surface area; SAED, selected area electron diffraction; SDS, sodium dodecyl sulphate; TEM, transmission electron microscopy; TPrA, tri-N-propylamine; VOCs, volatile organic compounds; WPE, wall plug efficiency.
